# Effects of betaine supplementation on dry matter intake, milk characteristics, plasma non-esterified fatty acids, and β-hydroxybutyric acid in dairy cattle: a meta-analysis

**DOI:** 10.1093/jas/skae241

**Published:** 2024-08-19

**Authors:** Muhammad I Malik, Muhammad Bilal, M Z Anwar, Talal Hassan, Muhammad A Rashid, Divine Tarla, Frank R Dunshea, Long Cheng

**Affiliations:** Department of Veterinary Sciences, University of Turin, 10095, Italy; Department of Animal Nutrition, University of Veterinary and Animal Sciences, Lahore 54000, Pakistan; Department of Livestock Production, PMAS, Arid Agriculture University, Rawalpindi 10370, Pakistan; Department of Veterinary Sciences, University of Turin, 10095, Italy; Department of Animal Nutrition, University of Veterinary and Animal Sciences, Lahore 54000, Pakistan; School of Agriculture, Food, and Ecosystem Sciences, Faculty of Science, The University of Melbourne, Dookie Campus, Victoria 3647, Australia; School of Agriculture, Food, and Ecosystem Sciences, Faculty of Science, The University of Melbourne, Parkville, Victoria 3010, Australia; School of Biology, Faculty of Biological Science, The University of Leeds, Leeds LS2 9JT, UK; School of Agriculture, Food, and Ecosystem Sciences, Faculty of Science, The University of Melbourne, Dookie Campus, Victoria 3647, Australia

**Keywords:** betaine, dairy cows, meta-analysis, milk fat, milk lactose, milk protein

## Abstract

Betaine supplementation in dairy cattle has gained attention due to its potential benefits to production and health as a methyl donor, which can play a crucial role in the metabolism of dairy cows. The objective of the current meta-analysis was to quantify the effects of betaine supplementation on milk production, composition, β-hydroxybutyric acid (BHBA), and non-esterified fatty acids (NEFA). A systematic literature search was carried out, all relevant studies were retrieved, and the meta-analysis was carried out. The mean difference (**MD**) for dry matter intake (DMI) using the random-effects model was 0.499 kg/d (*P* < 0.0001). The subgroup analysis indicated that supplementing betaine in heat-stressed cows increased DMI by 0.584 kg/d (*P* < 0.001), while in cows not exposed to heat stress, DMI was increased by 0.381 kg/d (*P* = 0.007). The energy-corrected milk (ECM) increased by 1.36 kg/d (*P* < 0.0001). The milk fat yield was significantly increased in betaine-supplemented cows (MD = 0.040 kg/d, 95% CI = 0.015 to 0.065). The milk protein yield (kg/d) (MD = 0.014, *P* = 0.138) was increased (MD = 0.035, *P* = 0.0005) by betaine supplementation. The lactose yield (kg/d) was also significantly higher (MD = 0.055, *P* = 0.020) in betaine-supplemented cows. The standardized mean difference (**SMD**) for NEFA (SMD = − 0.447, 95% CI = − 1.029 to 0.135, *P* = 0.114) and BHBA (SMD = − 0.130, 95% CI = − 0.491 to 0.234). In conclusion, the findings from this meta-analysis suggest that betaine supplementation positively influences DMI, ECM, milk fat yield, milk lactose yield, and milk protein yield. Subgroup analysis further indicated that the positive effects on DMI are greater in heat-stressed cows compared to those not exposed to heat stress. The analysis did not find significant effects on the levels of NEFA or BHBA, suggesting that betaine supplementation may not directly influence these metabolic parameters.

## Teaser

The supplementation of betaine led to increased dry matter intake and milk production in dairy cattle; this increase was more pronounced in heat-stressed cows.

## Introduction

Betaine, which is also known as tri-methyl glycine, is a derivative of amino acid and is naturally present in various plant and invertebrate species. One of the key physiological roles of betaine is its ability to regulate the balance of water in cells, thereby ensuring cellular integrity, and making it an important osmoregulation (Eklund et al., 2005). The reduction in ion pumping needed to maintain intracellular osmolarity in boars, consuming betaine can reduce endogenous heat production and this spared energy can be used for productive purposes (Suster et al., 2004). Additionally, it can act as a methyl group donor through *S*-adenosyl methionine (Lever and Slow, 2010). Here, betaine is involved in this process through the enzyme betaine-homocysteine methyltransferase in chicken (Kidd et al., 1997). As a methyl donor, betaine may spare methionine from being used as a methyl donor and improve the incorporation of methionine into milk protein (Peterson et al., 2012). The diets of dairy cows are mostly deficient in methionine (NASEM, 2021). Therefore, to meet the nutritional requirements, synthetic methionine is often provided (Watanabe, 2006). However, supplementation of synthetic methionine is expensive, and ruminant nutritionists often seek more cost-effective alternatives, such as higher protein diets or less expensive methionine alternatives. Supplementation of betaine may be one such alternative (Wang et al., 2020), which improves dry matter intake (DMI), milk yield in dairy cattle (Dunshea et al., 2019; Shah et al., 2020), and milk fat (Peterson et al., 2012). On the contrary, some studies have reported no effects of betaine supplementation in dairy cattle on dry matter intake (**DMI**), milk yield (Williams et al., 2021), or milk components (Davidson et al., 2008; Wang et al., 2019).

Betaine supplementation in lactating dairy cows increase in milk production in heat-stressed (**HS**) (Hall et al., 2016) and non-HS cows (Wang et al., 2010; Monteiro et al., 2017). Betaine can help alleviate the effects of HS in various animals, such as sheep (DiGiacomo et al., 2016) and beef cattle (DiGiacomo et al., 2014). In part, this may be due to the reduced ion pumping and heat production mentioned previously. Nonetheless, the impact of betaine supplementation on lactating dairy cows experiencing HS remains uncertain (Zhang et al., 2014; Hall et al., 2016). Additionally, it reduces plasma non-esterified acids (**NEFA**) and β-hydroxybutyric acid (**BHBA**) in non-HS cows (Wang et al., 2010). The most important limitation of previously published literature on dairy cattle and betaine is its availability in the rumen and its impact on rumen fermentation. The rumen protection of betaine and its availability could be beneficial for nutritional management as well as research for future guidelines for the application of betaine and its dosage. Most of the studies included in meta-analyses have not reported on rumen protection. Betaine supplementation is therefore a topic of interest in the field of animal nutrition. The objective of this study was, therefore, to perform a systematic review of published studies that investigated the impact of betaine supplementation on dairy cattle. Conducting a meta-analysis of betaine as a feed supplement on DMI, milk production, milk composition, NEFA, and BHBA, accounting for different potential important covariates, could be helpful in animal nutrition.

## Materials and Methods

### Search strategy

A comprehensive literature search was carried out on 9 March 2023, to retrieve the relevant titles of the articles. For the literature search, the following databases were searched: PUBMED (https://www.ncbi.nlm.nih.gov/pubmed/), AGRICOLA USDA, National Agriculture Library (https://search.nal.usda.gov/discovery/search?vid=01NAL_INST:MAIN), and Google Scholar (https://scholar.google.com/). The search terms for each database were “betaine and heifers”, “betaine and cow”, and “betaine and cattle”. For Google Scholar, filters were applied to restrict search titles. Additionally, articles were retrieved from the *Journal of Dairy Science* (https://www.journalofdairyscience.org/action/doSearch?text1=betaine&field1=AllField), with only a single keyword, “betaine”.

### Inclusion and exclusion criteria

**Figure 1.**
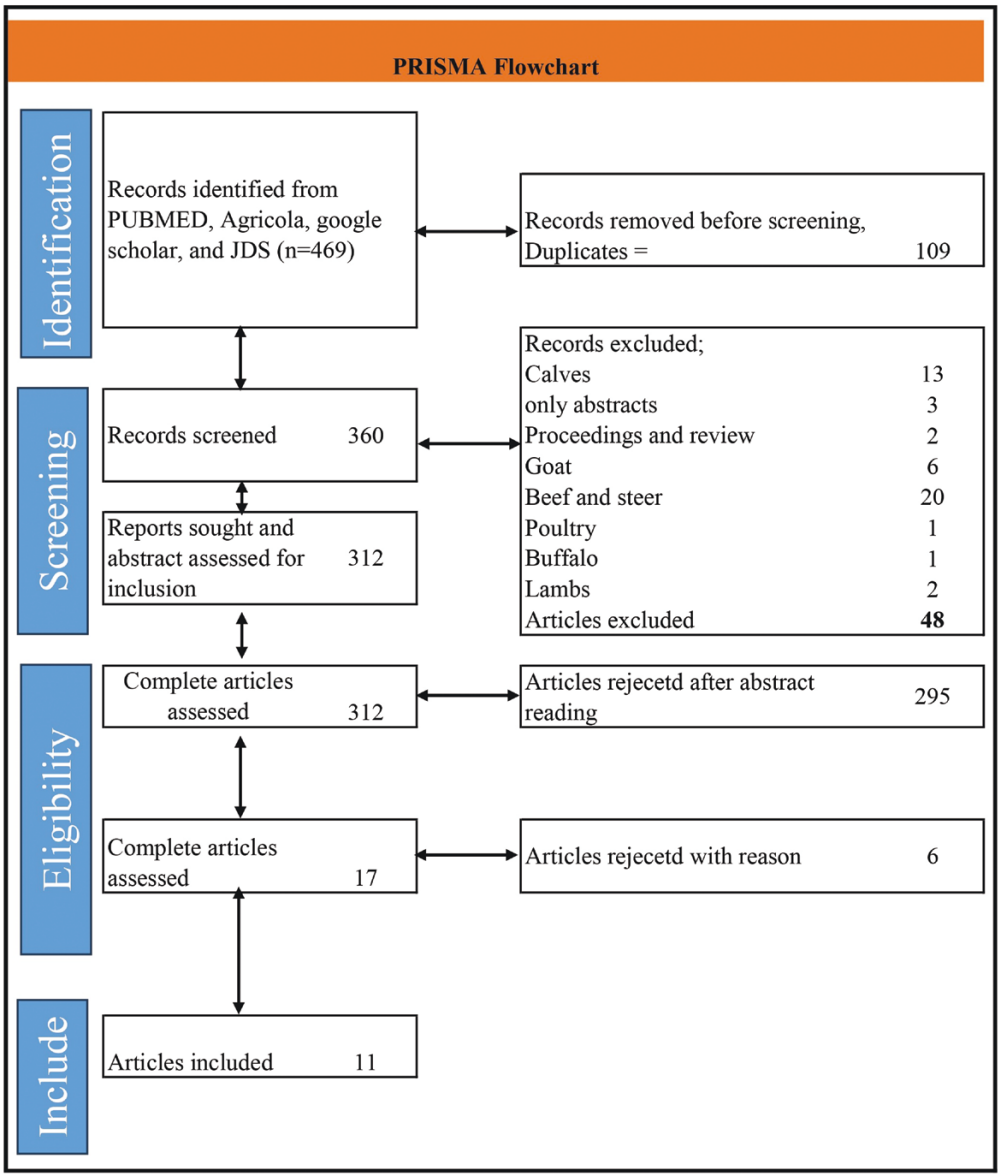
PRISMA flowchart and inclusion and exclusion details of the studies and database search details.


All relevant studies involving lactating cows (pregnant or nonpregnant) must have an experimental group supplemented with betaine and a control group. Studies were eligible if they evaluated DMI, milk production, milk components (fat, protein, and lactose), plasma NEFA, and BHBA for both control and treated cows. Restrictions were applied to the following publication types: conference abstracts, theses, books, and book chapters. We only included studies that were peer-reviewed, and articles published in English only. Once the initial title/abstract screening was completed, the full texts of the included studies from that stage were reviewed to determine if they should be included. Discrepancies were resolved by discussion, and the selection process was recorded in sufficient detail to complete a PRISMA flow diagram (Figure 1). The study characteristics and outcome data, study authors, year, number of cows (in the control and treated groups), mean, and standard deviation (**SD**) or standard error (**SE**) of the mean for outcome variables were collected from the studies included in the meta-analysis. The energy-corrected milk (ECM) was calculated using (Orth, 1992) :

$$\begin{array}{lll} ECM=\; & [\left(milk\;yield\times 0.327\right)+\left(fat\;yield\times 12.95\right)\; & +\left(protein\;yield\times 7.2\right)] \end{array}$$

The SE for ECM was calculated as


$$\begin{array}{lll} SE\;ECM=\; & \left(SE\;milk\times 0.327\right)+\left(SE\;fat\;yield\times 12.95\right)\; & +\left(SE\;protein\;yield\times 7.2\right). \end{array}$$

Milk fat, protein, and lactose data were extracted as total yield whenever the values were not reported, and then the means were calculated (e.g., fat yield = LSM for milk yield × LSM for milk fat%). An approximation was used to calculate the SE whenever it was not available for yields of milk components (Arshad et al., 2020). For example:


$$\begin{array}{l} SEM\;for\;protein=protein\;yield\sqrt{{\left(\frac{SE\;milk}{LSM\;milk}\right)}^{2}+{\left(\frac{SE\;protein\;\%\;}{LSM\;protein}\right)}^{2}} \end{array}$$

The feed efficiency was calculated as ECM/DMI for betaine-supplemented and control cows. FE data were analyzed using a linear mixed model approach. Betaine and the control were fixed factors, while studies were added as random effects. Data for the variables to be included in the meta-regression were also extracted from selected studies. The variables were body weight (**BW**) of the cows, proportion of concentrate*s* (% on DM basis) of the diet, crude protein percentage (**CP**), days in milk (**DIM**), experimental duration (days), net energy of lactation (**NE**_**L**_), dose of betaine supplementation, parity of the cows, and whether they were exposed to HS or not. The HS status of the studies depended on primary studies. If studies reported any heat stress condition, they were considered HS. Those studies that did not mention any environmental conditions or heat stress conditions were considered non-heat stress. In cases where the energy content of the diet was presented as metabolizable energy, NE_L_ was calculated as (NRC, 2001):


$$\begin{array}{l} NE_{L}\left(Mcal/kg\right)=\left[0.703\;\times ME\;\left(Mcal/kg\right)\;\text{-}\;0.19\right] \end{array}$$

The missing variable values or characters were imputed by the random forest imputation method. Random forest imputation is a method used for imputing missing values in a dataset. It involves using a machine learning algorithm to estimate the missing values based on the values of other variables in the dataset. The random forest algorithm is a type of decision tree algorithm that builds many individual decision trees and combines their predictions to make a final prediction. The imputation analysis was carried out by MICE package version 3.15.0 (Van and Groothuis, 2011) with the help of OpenMEE (Wallace et al., 2017).

### Data analysis

The analysis was carried out using the mean difference (**MD**) of the effect size (experiment and control) for DMI, milk production, and milk composition (milk fat, milk protein, and milk lactose content). For NEFA and BHBA, the effect size was calculated as standardized mean difference (SMD). The SMD is a statistical technique utilized in meta-analysis to compare and merge findings from diverse studies that use varying scales of measurement (Cohen, 2013). To determine the SMD, the treatment mean was subtracted from the control mean, then the result was divided by the pooled standard deviation (Andrade, 2020). A positive SMD meant that the treatment group had a higher mean than the control group, while a negative SMD indicated that the control group had a higher mean than the treatment group. Effect sizes were categorized as small, medium, and large at values of 0.2, 0.5, and 0.8, respectively (Cohen et al., 2013).

A random-effects model was fitted to the data, and subgroup analysis was carried out for cows supplemented with betaine. The subgroup was created based on whether cows were exposed to HS or not. The amount of heterogeneity (*τ*^2^) was estimated using the DerSimonian-Laird estimator (DerSimonian and Laird, 1986). In addition to the estimate of *τ*^2^, the *Q*-test for heterogeneity (Cochran, 1954), and the *I*^2^ statistic, the value of *I*^2^ was determined using the formula *I*^2^ = (*Q* − df/*Q*) × 100, where *Q* represents the χ^2^ statistic and its degree of freedom. Based on the *I*^2^ values obtained, the level of heterogeneity was classified according to Higgins and Thompson (2002) as follows: no heterogeneity, *I*^2^ ≤ 25%; low heterogeneity, 25% to 50%; moderate heterogeneity, 50% to 75%; and high heterogeneity > 75%. In case of heterogeneity (*I*^2^ > 50), a meta-regression was carried out to determine the source of the heterogeneity, and a prediction interval for the true outcomes was also provided (Riley et al., 2011). The confidence intervals were computed using the Knapp and Hartung adjustment method (Knapp and Hartung, 2003). For a moderator to be included in meta-regression, a threshold of a minimum of 4 studies per subset/subgroup for inclusion in the model was predefined according to the recommendation (Fu et al., 2011). The rank correlation test (Begg and Mazumdar, 1994) and the regression test (Sterne and Egger 2005), both employing the SE of observed outcomes as a predictor, are applied to assess funnel plot asymmetry to detect publication bias (Egger et al., 1997). The analysis was carried out using R version 4.1.3 (R Core Team, 2020) and the Metafor package version 4.1.3 (Viechtbauer, 2010). When effect sizes are dependent, perhaps because they are based on the same sample or obtained from the same set of researchers, it is inappropriate to use the standard two-level models. This is because they assume conditional independence of the effect sizes, meaning that no relationships among the effect sizes exist once controlling for the moderators in the model (Pastor and Lazowski, 2018). To address the dependence of effect sizes extracted from multiple studies, a three-level meta-analytical random effect model was employed. This model is a robust approach for handling such dependence, and it is particularly suitable when there is heterogeneity between studies. In this method, the effect sizes extracted from the same study were considered higher-level and nested, and the multilevel meta-analytical model was employed, which is the most appropriate model for such scenarios. By accounting for the varying levels of variation within and between studies, the multilevel meta-analysis technique can provide more precise estimates of treatment effects and aid in identifying the sources of heterogeneity (Cheung, 2014; Assink and Wibbelink, 2016). The meta-regression was carried out as random-effects model with Metafor package version 4.1.3 (Viechtbauer, 2010).

## Results

### Dry matter intake

**Figure 2.**
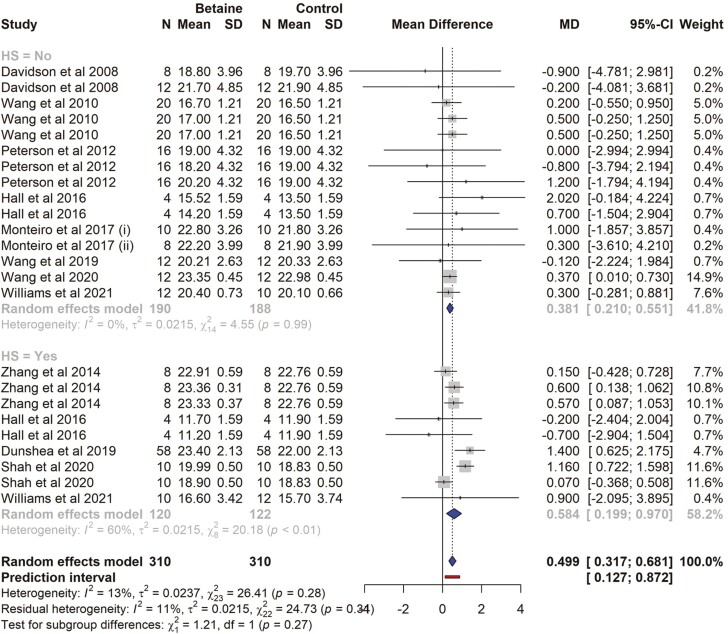
The forest plot from the random-effects meta-analysis showed the effect of betaine supplementation on dry matter intake (kg/d). A subgroup analysis was conducted to examine the effects on cows exposed to heat stress (HS = Yes) and those not exposed to heat stress (HS = No). The effect size was calculated as (betaine-control) using the mean difference (MD), where a negative value under the MD heading denotes a decrease in dry matter intake (kg/d) and a positive value denotes an increase. A dotted vertical line indicates the average effect size. The effect size of the associated study is shown as a grey box, and the confidence interval is shown as a black line that runs horizontally. The diamond reflects the average effect size of the subgroup, and the red line denotes the prediction interval. Additionally, the number of cows and standard deviation are indicated as *N* and SD, respectively.

**Figure 3.**
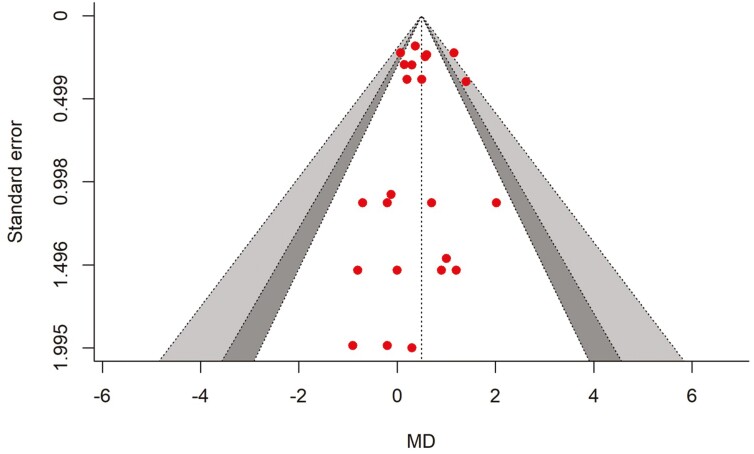
The contour-enhanced funnel plot. The symmetrical distribution of effect size around standard error of the study represents no evidence of publication bias in DMI meta-analysis.

There were 11 studies and a total of 24 effect sizes (*k* = 24) included in the DMI meta-analysis. The data collected and study characteristics included in the meta-analysis are presented in Supplementary Table S1. The observed MD ranged from − 0.900 to 2.020, with the majority of estimates being positive (71%). The estimated average MD was significantly higher in betaine treatment (MD* *= 0.499, 95% CI = 0.317 to 0.681, t = 5.66, *P* < 0.0001). The subgroup analysis indicated that when supplementing betaine in HS cows, the DMI was increased by 0.584 kg/d (*P* < 0.001, 95% CI = 0.021 to 0.969), while in cows not exposed to HS, the DMI was increased by 0.381 kg/d (*P* = 0.007, 95% CI = 0.209 to 0.551), between subgroups (HS vs non-HS) analysis, there was a no significant difference in the effect of betaine on DMI (*P* = 0.31). A forest plot showing the observed outcomes and the estimate based on the random-effects model is shown in Figure 2. According to the *Q*-test, there was no significant heterogeneity in the true outcomes (*Q* = 26.41, *P* = 0.282, *τ*^2^ = 0.023, *I*^2^ = 12.90%). At a 95% prediction interval, the true outcomes ranged from 0.127 to 0.872. The effect size for multilevel random-effects meta-analysis for DMI was significantly higher (MD = 0.497, *t* = 4.434, *P* = 0.0002, 95% CI = 0.265 to 0.730) in betaine-supplemented cows as compared to those of the control (Table 1). No publication bias was detected for DMI (Figure 3). The rank correlation and the regression test were also non-significant (*P* = 0.82 and 0.64, respectively).

**Table 1.** The summary statistics for random effects and multilevel random effects meta-analysis for betaine supplementation and its effects on DMI (kg/d)

Parameters	Estimate	SE	*t*-value	*P*-value	95% CI
Random effects MA	0.499	0.08	5.669	<0.0001	0.317, 0.681
Multilevel random MA	0.497	0.11	4.434	0.0002	0.265, 0.729

CI, confidence interval.

### Milk production (ECM)

**Figure 4.**
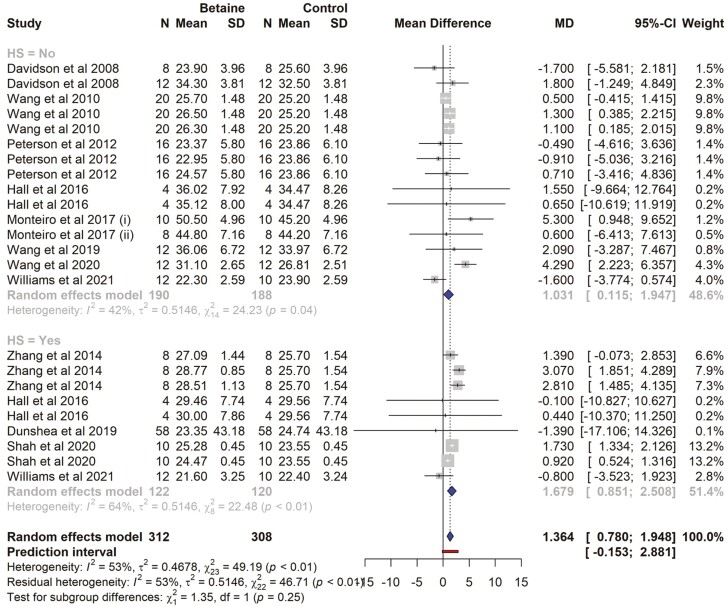
The forest plot from the random-effects meta-analysis showed the effect of betaine supplementation on energy-corrected milk production (kg/d). A subgroup analysis was conducted to examine the effects on cows exposed to heat stress (HS = Yes) and those not exposed to heat stress (HS = No). The effect size was calculated as (betaine-control) using the mean difference (MD), where a negative value under the MD heading denotes a decrease in milk production (kg/d) and a positive value denotes an increase. A dotted vertical line indicates the average effect size. The effect size of the associated study is shown as a grey box, and the confidence interval is shown as a black line that runs horizontally. The diamond reflects the average effect size of the subgroup, and the red line denotes the prediction interval. Additionally, the number of cows and standard deviation are indicated as *N* and SD, respectively.

There were 11 studies and a total of 24 effect sizes (*k* = 24) included in the milk production meta-analysis. The data collected and study characteristics included are presented in Supplementary Table S2. The observed mean differences ranged from − 1.70 to 5.30 kg/d, with the majority of estimates being positive (71%). The estimated average ECM was significantly higher in betaine-supplemented cows (MD = 1.36 kg/d, 95% CI = 0.779 to 1.948; *t* = 4.82, *P* < 0.0001). In subgroup analysis for HS cows, ECM was increased by 1.67 kg/d (*P* = 0.0004, 95% CI = 0.851 to 2.507), while for those cows not exposed to HS, ECM was increased by 1.03 kg/d (*P* = 0.018, 95% CI = 0.114 to 1.947). There was no significant difference (*P* = 0.25) between HS and non-HS subgroups (Figure 4). The effect size for multilevel random-effects meta-analysis for ECM was (MD = 1.36 kg/d, *t* = 2.46, *P* = 0.021, 95% CI = 0.219 to 2.515), significantly higher in betaine-supplemented cows compared to those of the control (Table 2). According to the *Q*-test, the true outcomes appeared to be heterogeneous (*Q* = 49.18, *P* = 0.001, *τ*^2^ = 0.467, *I*^2^ = 53.24%). The 95% prediction interval for the true outcomes was from −0.166 to 2.89. The overall heterogeneity contribution of each study is presented in the Baujat plot (Supplementary Figure S1). The highest contribution is shared by Shah et al. (2020).

**Table 2.** Effects of betaine supplementation on ECM (kg/d) production, random effects, multilevel random effects meta-analysis, and variables included in meta-analysis

Parameters	Estimate	SE	*t*-value	*P*-value	95% CI
Random effects MA	1.364	0.282	4.828	0.0001	0.779, 1.948
Multilevel random MA	1.367	0.554	2.464	0.021	0.219, 2.515
Variables					
BW	0.020	0.015	1.338	0.197	0.011, 0.052
DIM	−0.002	0.007	−0.359	0.723	−0.019, 0.013
Con	−0.029	0.043	−0.671	0.510	−0.121, 0.062
NE_L_	−6.572	5.253	−1.253	0.226	−17.58, 4.444
Exp duration	0.019	0.024	0.800	0.433	−0.031, 0.069
Dose	0.004	0.010	0.436	0.667	−0.017, 0.026
CPbetaine interaction	−−0.001	0.011	−0.125	0.899	−0.230, 0.020

CI, confidence interval, BW, body weight of the cows (kg), DIM, days in milk (days), Con, proportion of concentrates in the diet (%), Dose, dose of betaine supplementation in mg/d.

The meta-regression model indicated that the effect size was not influenced (*P* > 0.05) BW, DIM, proportion of concentrates in the diet, NE_L_, and dose of betaine supplementation (Table 2). No interactions (*P* = 0.89) were observed between supplemental betaine and dietary CP. The meta-regression model indicated that there are 48.73% of the total heterogeneity unexplained by moderators. The symmetrical distribution of effect sizes indicates the absences of publication bias (Supplementary Figure S2). Neither the rank correlation nor the regression test indicated funnel plot asymmetry (*P* = 0.861 and 0.50, respectively).

**Figure 5.**
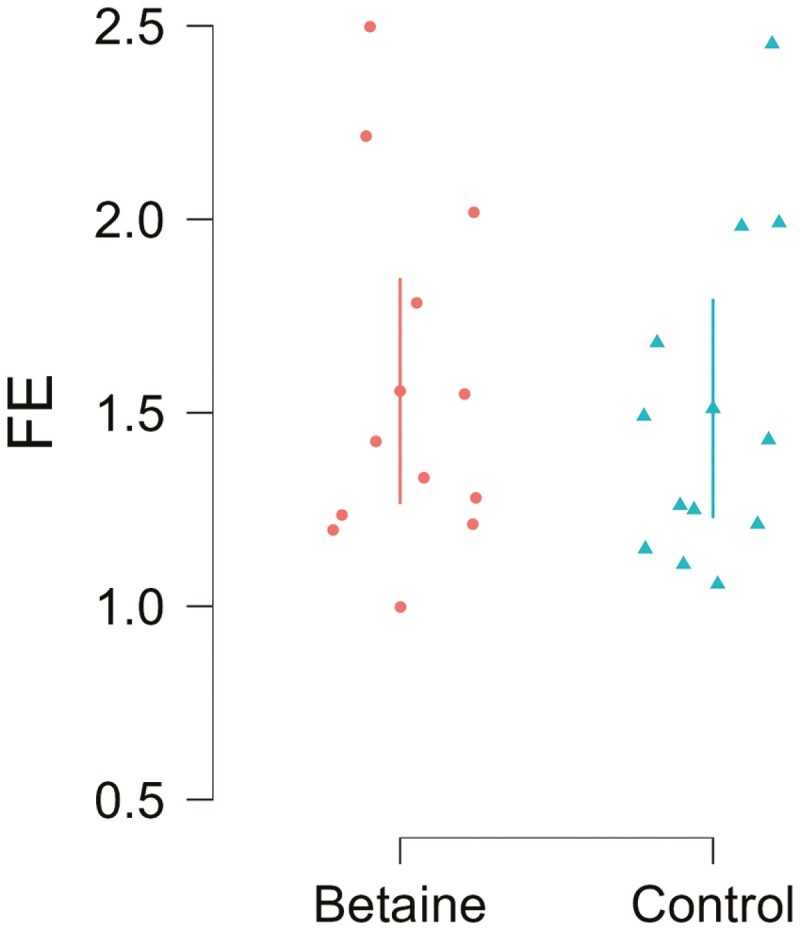
The feed efficiency (FE) calculated as ECM/DMI, the estimated means for betaine and control are 1.55, and 1.51, respectively.

The linear mixed model effects for feed efficiency (ECM/DMI) were also non-significant (*P* = 0.117). The average FE for betaine-supplemented and control cows was 1.55 and 1.51, respectively (Figure 5).

### Milk fat yield

**Figure 6.**
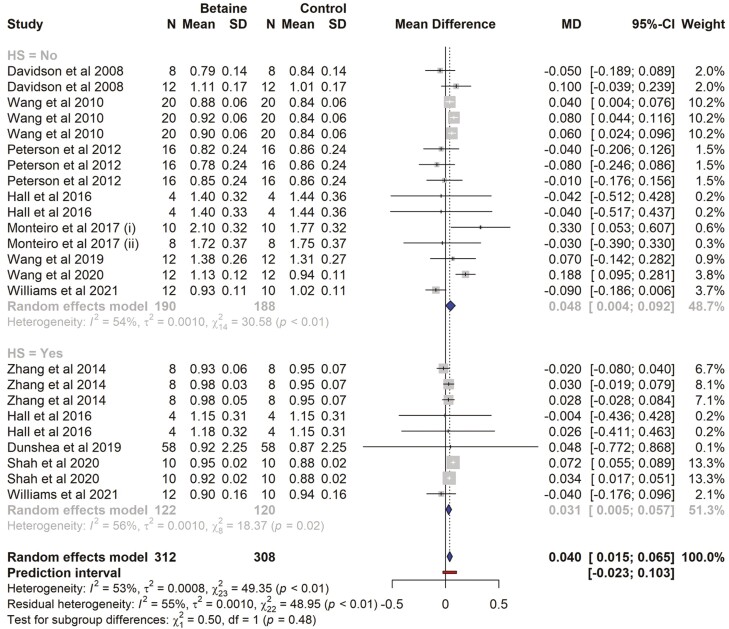
The forest plot from the random-effects meta-analysis showed the effect of betaine supplementation on milk fat yield kg per day. A subgroup analysis was conducted to examine the effects on cows exposed to heat stress (HS = Yes) and those not exposed to heat stress (HS = No). The effect size was calculated as (betaine-control) using the mean difference (MD), where a negative value under the MD heading denotes a decrease in milk fat yield (kg/d) and a positive value denotes an increase. A dotted vertical line indicates the average effect size. The effect size of the associated study is shown as a grey box, and the confidence interval is shown as a black line that runs horizontally. The diamond reflects the average effect size of the subgroup, and the red line denotes the prediction interval. Additionally, the number of cows and standard deviation are indicated as *N* and SD, respectively.

There were 11 studies and a total of 24 effect sizes (*k* = 24) included in the milk fat meta-analysis. The data collected and study characteristics included are presented in Supplementary Table S3. The MD observed ranged from −0.09 to 0.33, with the majority of estimates being positive (54%). Using the random-effects model, the estimated average mean difference was significantly higher in betaine-supplemented cows (MD = 0.040 kg/d, 95% CI = 0.015 to 0.065, *t* = 3.35; *P* = 0.022). In the subgroup analysis, milk fat content was not found to be different for cows under HS (MD = 0.031 kg/d, *P* = 0.091, CI = −0.005 to 0.067), while for cows in thermoneutral conditions, the milk fat content was significantly increased (MD = 0.047 kg/d, *P* = 0.013, CI = 0.0108 to 0.084) with betaine supplementation (Figure 6). There was no significant difference (*P* = 0.48) between the subgroups (HS vs. non-HS). The effect size for multilevel random-effects meta-analysis for milk fat yield (MD = 0.038 kg/d, *t* = 1.59, *P* = 0.124, 95% CI = −0.011 to 0.088) was also non-significant in betaine-supplemented cows as compared to those of the control. According to the Q-test, the true outcomes appeared to be heterogeneous (*Q* = 49.35, *P* = 0.0018, *τ*^2^ = 0.0003, *I*^2^ = 55%). The meta-regression model indicated that the effect size was influenced by the CP of the diet (estimate = −0.042, *P* = 0.002). The interactions between betaine supplementation and dietary CP were non-significant (*P* = 0.903). The NE_L_, proportion of concentrates in the diet, dose of betaine supplementation, experiment duration, and BW had no influence on effect size (*P* > 0.05; Table 3). Without variables, the heterogeneity (*I*^2^) was 55% in regression model (*I*^2^ = 0.00%), the 55% of total heterogeneity was associated with variables included in the meta-regression. There was no publication bias (Supplementary Figure S3). Both the rank correlation and the regression test for funnel plot asymmetry were non-significant (*P* = 0.601 and 0.385, respectively).

**Table 3.** Effects of betaine supplementation on milk fat yield (kg/d), random effects, multilevel random effects meta-analysis, and variables included in meta-analysis

Parameters	Estimate	SE	*t*-value	*P*-value	95% CI
Random effects MA	0.040	0.012	3.35	0.002	0.015, 0.064
Multilevel random MA	0.038	0.024	1.595	0.124	−0.011, 0.088
Variables					
CP	−0.042	0.012	−3.507	0.002	−0.067, −0.016
NE_L_	0.298	0.184	1.615	0.124	−0.091, 0.687
DIM	0.0003	0.000	−3.692	0.001	−0.000, −0.000
Con	−0.002	0.001	−1.698	0.107	−0.004, 0.000
Dose	0.0001	0.000	0.521	0.609	−0.000, 0.000
Exp duration	−0.001	0.000	1.655	0.116	−0.000, 0.002
BW	0.0006	0.000	1.866	0.079	−0.000, 0.000
CP*betaine interaction	0.0001	0.0004	0.121	0.903	−0.000, 0.000

CI, confidence interval, BW, body weight of the cows, DIM, days in milk, Con, proportion of concentrates in the diet, Dose, dose of betaine supplementation in mg/day.

### Milk lactose yield

**Figure 7.**
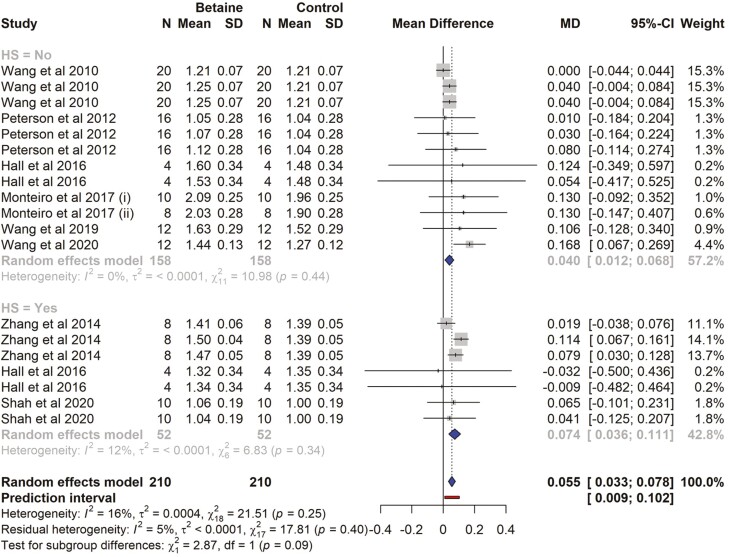
The forest plot from the random-effects meta-analysis showed the effect of betaine supplementation on milk lactose yield (kg/d). A subgroup analysis was conducted to examine the effects on cows exposed to heat stress (HS = Yes) and those not exposed to heat stress (HS = No). The effect size was calculated as (betaine-control) using the mean difference (MD), where a negative value under the MD heading denotes a decrease in milk lactose yield (kg/d) and a positive value denotes an increase. A dotted vertical line indicates the average effect size. The effect size of the associated study is shown as a grey box, and the confidence interval is shown as a black line that runs horizontally. The diamond reflects the average effect size of the subgroup, and the red line denotes the prediction interval. Additionally, the number of cows and standard deviation are indicated as *N* and SD, respectively.

There were 8 studies and a total of 19 effect sizes (*k* = 19) included in the milk lactose meta-analysis (Supplementary Table S4). The observed mean differences ranged from −0.032 to 0.168, with the majority of estimates being positive (84%). The estimated average mean difference (MD) was 0.055 (kg/d) (95% CI = 0.0332 to 0.077; *t* = 5.24, *P* < 0.0001) in betaine-supplemented cows (Table 4). The subgroup analysis revealed that cows exposed to HS had a significantly higher lactose yield (MD = 0.073 kg/d, *P* = 0.0001), similarly, cows not exposed to HS, lactose yield was significantly higher (MD = 0.039 kg/d, *P* = 0.006) by betaine supplementation (Figure 7). There was no significant difference (*P* = 0.09) between the subgroups (HS vs. non-HS). The effect size for multilevel random-effects meta-analysis for milk lactose yield was significantly higher (MD = 0.064 kg/d, *t* = 3.386, *P* = 0.003, 95% CI = 0.024 to 0.103) in betaine-supplemented cows as compared to those of the control. According to the *Q*-test, the true outcomes appeared to be non-heterogeneous (*Q* = 21.51, *P* = 0.254, *τ*^2^ = 0.0002, *I*^2^ = 16.3).

**Table 4.** Effects of betaine supplementation on milk lactose yield (kg/d), random effects, and multilevel random effects meta-analysis

Parameters	Estimate	SE	*t*-value	*P*-value	95% CI
Random effects MA	0.055	0.010	5.248	< 0.0001	0.033, 0.077
Multilevel Random MA	0.064	0.018	3.386	0.003	0.024, 0.103

CI, confidence interval.

Neither the rank correlation nor the regression test indicated funnel plot asymmetry (*P* = 0.438 and 0.527, respectively), as shown in Supplementary Figure S4.

### Milk protein yield

**Figure 8.**
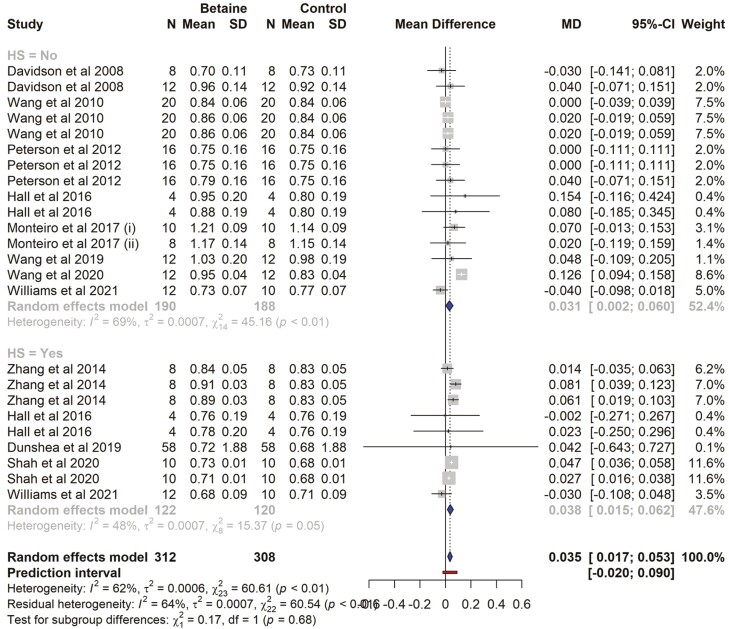
The forest plot from the random-effects meta-analysis showed the effect of betaine supplementation on milk protein yield (kg/d). A subgroup analysis was conducted to examine the effects on cows exposed to heat stress (HS = Yes) and those not exposed to heat stress (HS = No). The effect size was calculated as (betaine-control) using the mean difference (MD), where a negative value under the MD heading denotes a decrease in milk protein yield (kg/day) and a positive value denotes an increase. A dotted vertical line indicates the average effect size. The effect size of the associated study is shown as a grey box, and the confidence interval is shown as a black line that runs horizontally. The diamond reflects the average effect size of the subgroup, and the red line denotes the prediction interval. Additionally, the number of cows and standard deviation are indicated as *N* and SD, respectively.

There were 11 studies and a total of 24 effect sizes (*k* = 24) included in the milk protein meta-analysis (Supplementary Table S5). The observed mean differences ranged from −0.040 to 0.154. The estimated average MD for milk protein yield was significantly higher (MD = 0.035 kg/d, 95% CI = − 0.017 to 0.053; *t* = 4.015, *P* = 0.0005) in betaine supplemented cows. The subgroup analysis revealed that milk protein yield (kg/d) in both HS and non-HS cows was significantly higher (*P* = 0.007, *P* = 0.017, respectively) in betaine supplemented cows (MD = 0.038 and 0.031, respectively). The difference between the subgroups (HS vs. non-HS) was not significant, with *P* = 0.678 (Figure 8). The effect size for multilevel random-effects meta-analysis for milk protein yield (kg/d) was significantly higher (MD = 0.037, *t* = 2.386, *P* = 0.025, 95% CI = 0.005 to 0.070) in betaine-supplemented cows compared to those of the control (Table 5). According to the *Q*-test, the true outcomes appear to be heterogeneous (*Q* = 60.61, *P* < 0.0001, τ^2^ = 0.0006, *I*^2^ = 62.1). The meta-regression model indicated that the effect size was influenced by the CP of the diet (estimate = -0.024, *P* = 0.038). The interaction between betaine dose and CP level of the diet was non-significant (*P* = 0.360). The NEL, experimental duration, proportion of concentrates in the diet, dose of betaine supplementation, and BW had no influence on effect size (*P* > 0.05) (Table 3). Without variables, the heterogeneity (*I*^2^) was 62%. In the regression model (*I*^2^ = 27.15%), 34.85% of total heterogeneity was associated with variables included in the meta-regression. A funnel plot of the estimates is shown in Supplementary Figure S5. Neither the rank correlation nor the regression test indicated funnel plot asymmetry (*P* = 0.469 and *P* = 0.548, respectively).

**Table 5.** Summary of random effects and multilevel random effects meta-analysis for milk protein yield (kg/d)

Parameters	Estimate	SE	*t*-value	*P*-value	95% CI
Random effects MA	0.035	0.0087	4.015	0.0005	0.0170, 0.0532
Multilevel random MA	0.037	0.0157	2.386	0.025	-0.005, 0.070
Variables					
CP	−0.0246	0.011	−2.23	0.038	−0.0479, −0.0014
Con	0.0002	0.001	0.137	0.892	−0.0027, 0.0031
NE_L_	0.2213	0.202	1.095	0.288	−0.2049, 0.6474
Dose	−0.0002	0.0003	−0.699	0.493	−0.0008, 0.0004
DIM	0.0001	0.0002	0.290	0.775	−0.0004, 0.0005
Exp duration	0.0011	0.0007	1.517	0.147	−0.0004, 0.002
BW	0.0001	0.0004	0.206	0.838	−0.0008, 0.001
CP*betaine interaction	0.0003	0.0003	0.914	0.360	−0.0003, 0.0008

CI, confidence interval, BW, body weight of the cows (kg), DIM, days in milk (d), Con, proportion of concentrates in the diet (%), Dose, dose of betaine supplementation in mg/d.

### Plasma non esterified fatty acids

**Figure 9.**
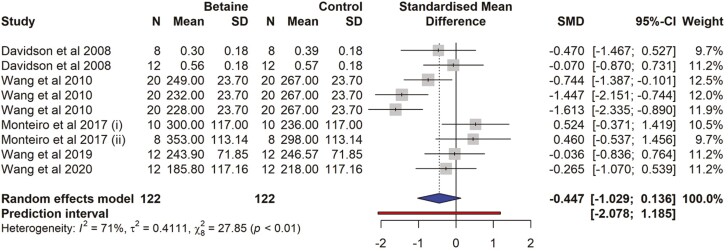
The forest plot from the random-effects meta-analysis showed the effect of betaine supplementation on plasma NEFA. The effect size was calculated as [(betaine-control)/pooled standard deviation] using the standardized mean difference (SMD), where a negative value under the SMD heading denotes a decrease in NEFA and a positive value denotes an increase. A dotted vertical line indicates the average effect size. The effect size of the associated study is shown as a grey box, and the confidence interval is shown as a black line that runs horizontally. The diamond reflects the average effect size of the subgroup, and the red line denotes the prediction interval. Additionally, the number of cows and standard deviation are indicated as *N* and SD, respectively.

There were four studies and a total of nine effect sizes (*k* = 9) included in NEFA meta-analysis (Supplementary Table S6). The observed SMD ranged from −1.612 to 0.523, with most estimates being negative (78%). The estimated average SMD (−0.447) was not significant (95% CI = − 1.029 to 0.135; *t* = − 1.769, *P* = 0.114; Figure 9). The effect size for multilevel random-effects meta-analysis for NEFA (SMD = -0.311, *t* = −0.847, *P* = 0.42, 95% CI = −1.159 to 0.536) was also non-significant in betaine-supplemented cows as compared to control (Table 6).

**Table 6.** Effects of betaine supplementation on blood NEFA, random effects, multilevel random effects model summary in meta-analysis

Parameters	Estimate	SE	*t*-value	*P*-value	95% CI
Random effects MA	−0.447	0.252	−1.769	0.114	−1.029, 0.135
Multilevel random MA	−0.311	0.368	−0.847	0.421	−1.159, 0.536

95% CI, confidence interval.

### Beta hydroxybutyric acid

**Figure 10.**
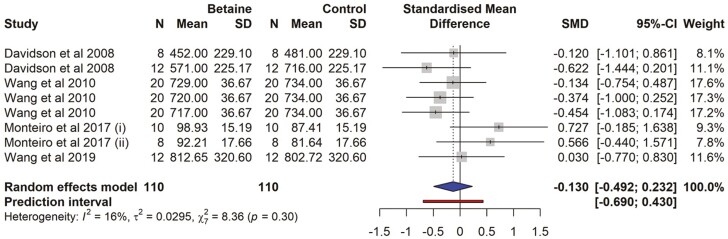
The forest plot from the random-effects meta-analysis showed the effect of betaine supplementation on BHBA. The effect size was calculated as [(betaine-control)/pooled standard deviation] using the standardized mean difference (SMD), where a negative value under the SMD heading denotes a decrease in BHBA and a positive value denotes an increase. A dotted vertical line indicates the average effect size. The effect size of the associated study is shown as a grey box, and the confidence interval is shown as a black line that runs horizontally. The diamond reflects the average effect size of the subgroup, and the red line denotes the prediction interval. Additionally, the number of cows and standard deviation are indicated as N and SD, respectively.

There were 4 studies and a total of 8 effect sizes (*k* = 8) included in the analysis (Supplementary Table S7). The observed SMD ranged from − 0.621 to 0.726, with most estimates being negative (62%). The estimated average SMD was not significant (SMD = −0.128 (95% CI = −0.491 to 0.234, *t*= −0.836, *P* = 0.43). For blood BHBA, there was no subgroup analysis because all effect sizes belonged to those cows not exposed to HS. The effect size for multilevel random-effects meta-analysis for blood BHBA (SMD = −0.054, *t* = −0.238, *P* = 0.83, 95% CI = −0.619 to 0.509) was non-significant in betaine-supplemented cows as compared to those of the control (Figure 10). According to the *Q*-test, there was no significant amount of heterogeneity in the true outcomes (*Q* = 8.44, *P* = 0.295, *τ*^2^ = 0.031, *I*^2^ = 16.20%). A 95% prediction interval for the true outcomes ranges from −0.690 to 0.430. No publication bias was detected (Supplementary Figure S6). The rank correlation and the regression tests were non-significant (*P* = 0.40 and *P* = 0.075, respectively).

## Discussion

A major finding from this meta-analysis is that betaine supplementation leads to increased DMI in both HS and non-HS cows. This increase aligns with findings from prior experiments where cows were provided with dietary betaine (Dunshea et al., 2019; Shah et al., 2020). There was no difference in the effect of betaine on DMI between HS and non-HS cows. The mechanism by which betaine improves DMI in cows is not fully understood. It is believed that betaine may affect the fermentation process in the rumen by either providing available ruminal nitrogen (Wang et al., 2010) or methyl groups (Cronje, 2016). The degradation of betaine by ruminal microbes has already been reported. Therefore, betaine could alter ruminal fermentation by serving as a source of either rumen-available nitrogen or methyl groups (Löest et al., 2017). Another mechanism responsible for an increase in DMI could be associated with the increase in ruminal nitrogen, which increases the production of certain beneficial microbes in the rumen. This can help improve DMI, neutral detergent fiber (**NDF**), and acid detergent fiber (**ADF**) total tract digestibility (Wang et al., 2010). The inclusion of betaine for dairy cows experiencing HS led to an increase in the total tract digestibility of dry matter (DM), organic matter (OM), CP, NDF, and ADF. These findings align with a previous study conducted by Wang et al. (2010), which reported similar improvements in nutrient digestibility when betaine was added to the diet. This positive effect could be attributed to betaine’s properties as an osmolyte and methyl donor, which help mitigate HS, maintain optimal rumen pH, and enhance the microbial composition in the rumen (Eklund et al., 2006). The limitation of the current meta-analysis lies with the data availability in primary studies regarding betaine supplementation, rumen protection, its availability in the rumen, and bypass contents after protection. In an in vitro experiment (Löest et al., 2001), 63% and 94% of the betaine from feed-grade betaine and concentrated beet molasses (betaine source), respectively, disappeared after 24 h of incubation in the ruminal contents of steers fed with a high-grain diet. There are similar findings reported in steers fed a high-grain diet (Mitchell et al., 1979). The ruminal degradation of betaine would suggest that the effects of supplemental betaine could be associated with an increase in DMI and milk production.

The ECM production was improved in betaine-supplemented cows. The effect sizes for HS and non-HS cows were 1.67 and 1.03 kg/d, respectively. Previous studies have demonstrated that betaine supplementation can improve milk production in cows (Wang et al., 2020). The increase in milk production could be associated with betaine, because betaine provides a methyl group for the conversion of homocysteine to methionine (McDevitt et al., 2000). Methionine has a direct influence on milk production and milk components in dairy cattle (Zanton et al., 2014). Another potential mechanism for an increase in milk production could be an increase in DMI in cows supplemented with betaine. There was a numerical difference in the milk production response to betaine between HS and non-HS cows. The HS cows produced 0.64 kg more milk per day compared to non-HS cows. We believe this difference could be associated with a similar increase in DMI. The betaine supplemented HS cows also consumed 0.20 kg DM more compared to non-HS cows that were supplemented with betaine. Heat stress typically reduces DMI in dairy cows (Dunshea et al., 2013) to reduce endogenous heat production. The osmoprotective action of betaine results in a reduction in ion pumping needed to maintain intracellular osmolarity with a subsequent decrease in heat production. This decrease in heat production in betaine-supplemented cows could mean that the HS-induced reduction in DMI may not be as severe as it is in non-supplemented cows. With respect to milk composition, only milk lactose content was increased in HS cows supplemented with betaine (0.064% effect size). This increase might have been caused using betaine as an HS-reducing agent in cows, which, in turn, increased feed intake and milk yield. Heat stress also leads to changes in milk quality, mainly by reducing milk lactose and milk protein levels (Ravagnolo and Misztal, 2000). The increase in milk fat yield because of betaine supplementation in lactating dairy cows (Wang et al., 2010) could be associated with the conversion of betaine into acetate by microorganisms (Mitchell et al., 1979), and acetate is directly correlated with milk fat (Bauman and Griinari, 2003). Additionally, it improves the digestibility of organic matter, crude protein, neutral detergent fiber, and acid detergent fiber (Monteiro et al., 2017). The findings of the current meta-analysis indicate that an increase in the CP of the diet decreased in-milk protein yield and milk fat yield in betaine-supplemented cows. This could be associated with feeding high protein (mean = 17.30%, median = 17.55%), which consequently decreased N efficiency (Broderick, 2003), high protein diets can result in decreased milk N efficiency, increased manure N excretion, and reduced profitability for dairy producers (Colmenero and Broderick, 2006).

The NEFA decreased numerically with betaine supplementation. The effect size was SMD = −0.447 which is generally considered to be almost a medium effect. The plasma NEFA can serve as an indicator of energy balance and the mobilization of energy (Canfield and Butler, 1991). In the present meta-analysis, betaine supplementation resulted in a non-significant reduction in plasma NEFA levels, suggesting an improvement in energy availability. Plasma NEFA concentrations are negatively related to energy balance in lactating dairy cows (Sechen et al., 1990) and so the reduction in NEFA could be due to an increase in DMI in dairy cows supplemented with betaine. We suggest that supplementation of betaine and its effects on plasma NEFA concentration in dairy cattle could be a topic of interest because there are only four studies with nine effect sizes. The non-significant effect size could be associated with the inability of the meta-analysis to detect potential effect sizes, due to the limited number of studies available. There is, therefore, a disparity in the response of plasma NEFA to betaine supplementation observed between the present meta-analysis and the study by Davidson et al. (2008). This could be attributed to the difference in DIM and lactation stages of the cows utilized.

## Conclusion

The findings of this meta-analysis suggest that betaine supplementation positively influences DMI, milk production, milk fat yield, milk lactose yield, and milk protein yield. Subgroup analysis further indicates that the positive effects are more pronounced in heat-stressed cows compared to those not exposed to HS. While the analysis did not find significant effects on plasma NEFA or BHBA. Overall, these findings support the potential benefits of betaine supplementation in dairy cattle, particularly in improving DMI, milk production, and milk composition.

## Supplementary Material

skae241_suppl_Supplementary_Tables_S1-S7_Figures_S1-S6

## Abbreviations

ADF: acid detergent fiber
BHBA: β-hydroxybutyric acid
BW: body weight
CP: crude protein
DIM: days in milk
DMI: dry matter intake
ECM: energy-corrected milk
HS: heat-stressed
MD: mean difference
NDF: neutral detergent fiber
NE_L_: net energy of lactation
SMD: standardized mean difference
