# Supplementary material for: Effects of betaine supplementation on dry matter intake, milk characteristics, plasma non-esterified fatty acids, and β-hydroxybutyric acid in dairy cattle: a meta-analysis
Source: J Anim Sci. 2024 Aug 19;102:skae241. doi: 10.1093/jas/skae241 (PMC11398911; doi:10.1093/jas/skae241)
Supplement: skae241_suppl_Supplementary_Tables_S1-S7_Figures_S1-S6 [file skae241_suppl_supplementary_tables_s1-s7_figures_s1-s6.docx]

Supplementary table 1

Raw data and variables available for dry matter intake meta-analysis

|  | Betaine | | | Control | | | Variables | | | | | | | | |
| --- | --- | --- | --- | --- | --- | --- | --- | --- | --- | --- | --- | --- | --- | --- | --- |
| Authors and Years | N | Mean | SD | N | Mean | SD | Concentrate % | CP % | NE_L_ | Dose g/day | HS | Parity | DIM | Exp Duration | BW kg |
| Davidson et al 2008 | 8 | 18.80 | 3.96 | 8 | 19.70 | 3.96 | 59.30 | 17.60 | 1.61 | 45 | No | PP | 28 | 63 | **576** |
| Davidson et al 2008 | 12 | 21.70 | 4.85 | 12 | 21.90 | 4.85 | 59.30 | 17.60 | 1.61 | 45 | No | MP | 28 | 63 | **676** |
| Wang et al 2010 | 20 | 16.70 | 1.21 | 20 | 16.50 | 1.21 | 50.00 | 16.10 | 1.58 | 50 | No | MP | 88 | 15 | 597 |
| Wang et al 2010 | 20 | 17.00 | 1.21 | 20 | 16.50 | 1.21 | 50.00 | 16.10 | 1.58 | 100 | No | MP | 88 | 15 | 597 |
| Wang et al 2010 | 20 | 17.00 | 1.21 | 20 | 16.50 | 1.21 | 50.00 | 16.10 | 1.58 | 150 | No | MP | 88 | 15 | 597 |
| Peterson et al 2012 | 16 | 19.00 | 4.32 | 16 | 19.00 | 4.32 | 63.30 | 18.00 | 1.61 | 25 | No | **MP** | 126 | 16 | 623 |
| Peterson et al 2012 | 16 | 18.20 | 4.32 | 16 | 19.00 | 4.32 | 63.30 | 18.00 | 1.61 | 50 | No | **MP** | 126 | 16 | 623 |
| Peterson et al 2012 | 16 | 20.20 | 4.32 | 16 | 19.00 | 4.32 | 63.30 | 18.00 | 1.61 | 100 | No | **MP** | 126 | 16 | 623 |
| Zhang et al 2014 | 8 | 22.91 | 0.59 | 8 | 22.76 | 0.59 | 55.80 | 16.90 | 1.48 | 10 | Yes | Mix | 101 | 56 | 576 |
| Zhang et al 2014 | 8 | 23.36 | 0.31 | 8 | 22.76 | 0.59 | 55.80 | 16.90 | 1.48 | 15 | Yes | Mix | 101 | 56 | 576 |
| Zhang et al 2014 | 8 | 23.33 | 0.37 | 8 | 22.76 | 0.59 | 55.80 | 16.90 | 1.48 | 20 | Yes | Mix | 101 | 56 | 576 |
| Hall et al 2016 | 4 | 11.70 | 1.59 | 4 | 11.90 | 1.59 | 34.98 | 19.51 | 1.74 | 34 | Yes | MP | 101 | 28 | 606 |
| Hall et al 2016 | 4 | 11.20 | 1.59 | 4 | 11.90 | 1.59 | 34.98 | 19.51 | 1.74 | 71 | Yes | MP | 101 | 28 | 606 |
| Hall et al 2016 | 4 | 15.52 | 1.59 | 4 | 13.50 | 1.59 | 34.98 | 19.51 | 1.74 | 34 | No | MP | 101 | 28 | 606 |
| Hall et al 2016 | 4 | 14.20 | 1.59 | 4 | 13.50 | 1.59 | 34.98 | 19.51 | 1.74 | 71 | No | MP | 101 | 28 | 606 |
| Monteiro et al 2017 (i) | 10 | 22.80 | 3.26 | 10 | 21.80 | 3.26 | 59.06 | 15.30 | 1.65 | 2031 | No | MP | 0 | 56 | 724 |
| Monteiro et al 2017 (ii) | 8 | 22.20 | 3.99 | 8 | 21.90 | 3.99 | 59.06 | 15.30 | 1.65 | 1978 | No | MP | 0 | 56 | 676 |
| Dunshea et al 2019 | 58 | 23.40 | 2.13 | 58 | 22.00 | 2.13 | **63.30** | **17.60** | **1.61** | 15 | Yes | **MP** | **0** | 22 | 617 |
| Wang et al 2019 | 12 | 20.21 | 2.63 | 12 | 20.33 | 2.63 | 47.21 | 17.49 | 1.63 | 20 | No | MP | 0 | 70 | **676** |
| Shah et al 2020 | 10 | 19.99 | 0.50 | 10 | 18.83 | 0.50 | 49.46 | 14.88 | 1.30 | 15 | Yes | MP | **0** | 60 | 550 |
| Shah et al 2020 | 10 | 18.90 | 0.50 | 10 | 18.83 | 0.50 | 49.46 | 14.88 | 1.30 | 30 | Yes | MP | **101** | 60 | 550 |
| Wang et al 2020 | 12 | 23.35 | 0.45 | 12 | 22.98 | 0.45 | 38.90 | 15.25 | 1.55 | 20 | No | MP | **101** | 56 | **606** |
| Williams et al 2021 | 12 | 20.40 | 0.73 | 10 | 20.10 | 0.66 | 34.01 | 19.17 | 1.64 | 16 | No | MP | 216 | 35 | 566 |
| Williams et al 2021 | 10 | 16.60 | 3.42 | 12 | 15.70 | 3.74 | 34.01 | 19.17 | 1.64 | 16 | Yes | MP | 216 | 35 | 566 |

N, number of cows, M, mean (DMI kg /day), SD, standard deviation, CP, crude protein of diet fed to cows (%), NE_L_, net energy of lactation, HS, cows exposed to heat stress or not, parity, parity of the cows, MP, multiparous, PP, primiparous, Mix, Both MP and PP, DIM, days in milk, exp duration (days), duration of the experiment, BW (kg), body weight of the cows. The DIM 0 indicates parturition days.

Note: Value in the table in blue and bold format were not reported in studies (missing value), we generated these values by random forest algorithm.

Supplementary table 2

Raw data for energy corrected milk production meta-analysis and variables available for analysis

|  | Betaine | | | Control | | | Variables | | | | | | | | |
| --- | --- | --- | --- | --- | --- | --- | --- | --- | --- | --- | --- | --- | --- | --- | --- |
| Authors and Years | N | Mean | SD | N | Mean | SD | Concentrate% | CP % | NE_L_ | Dose g/day | HS | Parity | DIM | Exp Duration | BW kg |
| Davidson et al 2008 | 8 | 23.9 | 3.960 | 8 | 25.6 | 3.960 | 59.30 | 17.60 | 1.61 | 45 | No | PP | 28 | 63 | **576** |
| Davidson et al 2008 | 12 | 34.3 | 3.811 | 12 | 32.5 | 3.811 | 59.30 | 17.60 | 1.61 | 45 | No | MP | 28 | 63 | **676** |
| Wang et al 2010 | 20 | 25.7 | 1.476 | 20 | 25.2 | 1.476 | 50.00 | 16.10 | 1.58 | 50 | No | MP | 88 | 15 | 597 |
| Wang et al 2010 | 20 | 26.5 | 1.476 | 20 | 25.2 | 1.476 | 50.00 | 16.10 | 1.58 | 100 | No | MP | 88 | 15 | 597 |
| Wang et al 2010 | 20 | 26.3 | 1.476 | 20 | 25.2 | 1.476 | 50.00 | 16.10 | 1.58 | 150 | No | MP | 88 | 15 | 597 |
| Peterson et al 2012 | 16 | 23.37 | 5.800 | 16 | 23.86 | 6.104 | 63.30 | 18.00 | 1.61 | 25 | No | **MP** | 126 | 16 | 623 |
| Peterson et al 2012 | 16 | 22.95 | 5.800 | 16 | 23.86 | 6.104 | 63.30 | 18.00 | 1.61 | 50 | No | **MP** | 126 | 16 | 623 |
| Peterson et al 2012 | 16 | 24.57 | 5.800 | 16 | 23.86 | 6.104 | 63.30 | 18.00 | 1.61 | 100 | No | **MP** | 126 | 16 | 623 |
| Zhang et al 2014 | 8 | 27.09 | 1.442 | 8 | 25.7 | 1.541 | 55.80 | 16.90 | 1.48 | 10 | Yes | Mix | 101 | 56 | 576 |
| Zhang et al 2014 | 8 | 28.77 | 0.849 | 8 | 25.7 | 1.541 | 55.80 | 16.90 | 1.48 | 15 | Yes | Mix | 101 | 56 | 576 |
| Zhang et al 2014 | 8 | 28.51 | 1.131 | 8 | 25.7 | 1.541 | 55.80 | 16.90 | 1.48 | 20 | Yes | Mix | 101 | 56 | 576 |
| Hall et al 2016 | 4 | 29.46 | 7.740 | 4 | 29.56 | 7.740 | 34.98 | 19.51 | 1.74 | 34 | Yes | MP | 101 | 28 | 606 |
| Hall et al 2016 | 4 | 30 | 7.860 | 4 | 29.56 | 7.740 | 34.98 | 19.51 | 1.74 | 71 | Yes | MP | 101 | 28 | 606 |
| Hall et al 2016 | 4 | 36.02 | 7.920 | 4 | 34.47 | 8.260 | 34.98 | 19.51 | 1.74 | 34 | NO | MP | 101 | 28 | 606 |
| Hall et al 2016 | 4 | 35.12 | 8.000 | 4 | 34.47 | 8.260 | 34.98 | 19.51 | 1.74 | 71 | NO | MP | 101 | 28 | 606 |
| Monteiro et al 2017 (i) | 10 | 50.5 | 4.965 | 10 | 45.2 | 4.965 | 59.06 | 15.30 | 1.65 | 100 | NO | MP | 0 | 56 | 724 |
| Monteiro et al 2017 (ii) | 8 | 44.8 | 7.156 | 8 | 44.2 | 7.156 | 59.06 | 15.30 | 1.65 | 100 | NO | MP | 0 | 56 | 676 |
| Dunshea et al 2019 | 58 | 23.35 | 43.181 | 58 | 24.74 | 43.181 | **63.30** | **17.60** | **1.61** | 15 | Yes | **MP** | **0** | 22 | 617 |
| Wang et al 2019 | 12 | 36.06 | 6.720 | 12 | 33.97 | 6.720 | 47.21 | 17.49 | 1.63 | 20 | NO | MP | 0 | 70 | **676** |
| Shah et al 2020 | 10 | 25.28 | 0.452 | 10 | 23.55 | 0.452 | 49.46 | 14.88 | 1.30 | 15 | Yes | MP | **0** | 60 | 550 |
| Shah et al 2020 | 10 | 24.47 | 0.452 | 10 | 23.55 | 0.452 | 49.46 | 14.88 | 1.30 | 30 | Yes | MP | **101** | 60 | 550 |
| Wang et al 2020 | 12 | 31.1 | 2.650 | 12 | 26.81 | 2.515 | 38.90 | 15.25 | 1.55 | 20 | NO | MP | **101** | 56 | **606** |
| Williams et al 2021 | 12 | 22.3 | 2.591 | 10 | 23.9 | 2.590 | 34.01 | 19.17 | 1.64 | 16 | NO | MP | 216 | 35 | 566 |
| Williams et al 2021 | 12 | 21.6 | 3.246 | 10 | 22.4 | 3.244 | 34.01 | 19.17 | 1.64 | 16 | Yes | MP | 216 | 35 | 566 |

N, number of cows, M, mean (energy milk production kg/day), SD, standard deviation, Cp, crude protein of diet fed to cows (%), NE_L_, net energy of lactation, HS, cows exposed to heat stress or not, parity, parity of the cows, MP, multiparous, PP, primiparous, Mix, Both MP and PP, DIM, days in milk (days), exp duration, duration of the experiment (days), BW, body weight of the cows (kg). The DIM 0 indicates parturition days.

Note: Value in the table in blue and bold format were not reported in studies (missing value), we generated these values by random forest algorithm.

Supplementary table 3

Raw data for milk fat yield (kg/day) meta-analysis and variables available for analysis

|  | Betaine | | | Control | | | Variables | | | | | | | | |
| --- | --- | --- | --- | --- | --- | --- | --- | --- | --- | --- | --- | --- | --- | --- | --- |
| Authors and Years | N | M | SD | N | M | SD | Concentrate% | CP % | NE_L_ | Dose mg/day | HS | Parity | DIM | Exp Duration | BW |
| Davidson et al 2008 | 8 | 0.79 | 0.14 | 8 | 0.84 | 0.14 | 59.30 | 17.60 | 1.61 | 45 | No | PP | 28 | 63 | **576** |
| Davidson et al 2008 | 12 | 1.11 | 0.17 | 12 | 1.01 | 0.17 | 59.30 | 17.60 | 1.61 | 45 | No | MP | 28 | 63 | **676** |
| Wang et al 2010 | 20 | 0.88 | 0.06 | 20 | 0.84 | 0.06 | 50.00 | 16.10 | 1.58 | 50 | No | MP | 88 | 15 | 597 |
| Wang et al 2010 | 20 | 0.92 | 0.06 | 20 | 0.84 | 0.06 | 50.00 | 16.10 | 1.58 | 100 | No | MP | 88 | 15 | 597 |
| Wang et al 2010 | 20 | 0.9 | 0.06 | 20 | 0.84 | 0.06 | 50.00 | 16.10 | 1.58 | 150 | No | MP | 88 | 15 | 597 |
| Peterson et al 2012 | 16 | 0.82 | 0.24 | 16 | 0.86 | 0.24 | 63.30 | 18.00 | 1.61 | 25 | No | **MP** | 126 | 16 | 623 |
| Peterson et al 2012 | 16 | 0.78 | 0.24 | 16 | 0.86 | 0.24 | 63.30 | 18.00 | 1.61 | 50 | No | **MP** | 126 | 16 | 623 |
| Peterson et al 2012 | 16 | 0.85 | 0.24 | 16 | 0.86 | 0.24 | 63.30 | 18.00 | 1.61 | 100 | No | **MP** | 126 | 16 | 623 |
| Zhang et al 2014 | 8 | 0.93 | 0.06 | 8 | 0.95 | 0.07 | 55.80 | 16.90 | 1.48 | 10 | Yes | Mix | 101 | 56 | 576 |
| Zhang et al 2014 | 8 | 0.98 | 0.03 | 8 | 0.95 | 0.07 | 55.80 | 16.90 | 1.48 | 15 | Yes | Mix | 101 | 56 | 576 |
| Zhang et al 2014 | 8 | 0.98 | 0.05 | 8 | 0.95 | 0.07 | 55.80 | 16.90 | 1.48 | 20 | Yes | Mix | 101 | 56 | 576 |
| Hall et al 2016 | 4 | 1.15 | 0.31 | 4 | 1.15 | 0.31 | 34.98 | 19.51 | 1.74 | 34 | Yes | MP | 101 | 28 | 606 |
| Hall et al 2016 | 4 | 1.18 | 0.32 | 4 | 1.15 | 0.31 | 34.98 | 19.51 | 1.74 | 71 | Yes | MP | 101 | 28 | 606 |
| Hall et al 2016 | 4 | 1.4 | 0.32 | 4 | 1.44 | 0.36 | 34.98 | 19.51 | 1.74 | 34 | No | MP | 101 | 28 | 606 |
| Hall et al 2016 | 4 | 1.4 | 0.33 | 4 | 1.44 | 0.36 | 34.98 | 19.51 | 1.74 | 71 | No | MP | 101 | 28 | 606 |
| Monteiro et al 2017 (i) | 10 | 2.1 | 0.32 | 10 | 1.77 | 0.32 | 59.06 | 15.30 | 1.65 | 100 | No | MP | 0 | 56 | 724 |
| Monteiro et al 2017 (ii) | 8 | 1.72 | 0.37 | 8 | 1.75 | 0.37 | 59.06 | 15.30 | 1.65 | 100 | No | MP | 0 | 56 | 676 |
| Dunshea et al 2019 | 58 | 0.92 | 2.25 | 58 | 0.87 | 2.25 | **63.30** | **17.60** | **1.61** | 15 | Yes | **MP** | **0** | 22 | 617 |
| Wang et al 2019 | 12 | 1.38 | 0.26 | 12 | 1.31 | 0.27 | 47.21 | 17.49 | 1.63 | 20 | No | MP | 0 | 70 | **676** |
| Shah et al 2020 | 10 | 0.96 | 0.02 | 10 | 0.88 | 0.02 | 49.46 | 14.88 | 1.30 | 15 | Yes | MP | **0** | 60 | 550 |
| Shah et al 2020 | 10 | 0.92 | 0.02 | 10 | 0.88 | 0.02 | 49.46 | 14.88 | 1.30 | 30 | Yes | MP | **101** | 60 | 550 |
| Wang et al 2020 | 12 | 1.13 | 0.12 | 12 | 0.94 | 0.11 | 38.90 | 15.25 | 1.55 | 20 | No | MP | **101** | 56 | **606** |
| Williams et al 2021 | 12 | 0.93 | 0.11 | 10 | 1.02 | 0.11 | 34.01 | 19.17 | 1.64 | 16 | No | MP | 216 | 35 | 566 |
| Williams et al 2021 | 12 | 0.9 | 0.16 | 10 | 0.94 | 0.16 | 34.01 | 19.17 | 1.64 | 16 | Yes | MP | 216 | 35 | 566 |

N, number of cows, M, mean (milk fat yield kg/day), SD, standard deviation, Cp, crude protein of the diet fed to cows (%), NE_L_, net energy of lactation, HS, cows exposed to heat stress or not, parity, parity of the cows, MP, multiparous, PP, primiparous, Mix, Both MP and PP, DIM, days in milk (days), exp duration, duration of the experiment (days), BW, body weight of the cows (kg). The DIM 0 indicates parturition days

Note: Value in the table in blue and bold format were not reported in studies (missing value), we generated these values by random forest algorithm.

Supplementary table 4

Raw data for milk lactose yield (kg/day) meta-analysis and variables available for analysis

| Authors and Years | Betaine | | | Control | | | Variables | | | | | | | | |
| --- | --- | --- | --- | --- | --- | --- | --- | --- | --- | --- | --- | --- | --- | --- | --- |
|  | N | M | SD | N | M | SD | Concentrate% | CP % | NE_L_ | Dose mg/day | HS | Parity | DIM | Exp Duration | BW |
| Wang et al 2010 | 20 | 1.21 | 0.07 | 20 | 1.21 | 0.07 | 50 | 16.1 | 1.58 | 50 | No | MP | 88 | 15 | 597 |
| Wang et al 2010 | 20 | 1.25 | 0.07 | 20 | 1.21 | 0.07 | 50 | 16.1 | 1.58 | 100 | No | MP | 88 | 15 | 597 |
| Wang et al 2010 | 20 | 1.25 | 0.07 | 20 | 1.21 | 0.07 | 50 | 16.1 | 1.58 | 150 | No | MP | 88 | 15 | 597 |
| Peterson et al 2012 | 16 | 1.05 | 0.28 | 16 | 1.04 | 0.28 | 63.3 | 18 | 1.61 | 25 | No | **MP** | 126 | 16 | 623 |
| Peterson et al 2012 | 16 | 1.07 | 0.28 | 16 | 1.04 | 0.28 | 63.3 | 18 | 1.61 | 50 | No | **MP** | 126 | 16 | 623 |
| Peterson et al 2012 | 16 | 1.12 | 0.28 | 16 | 1.04 | 0.28 | 63.3 | 18 | 1.61 | 100 | No | **MP** | 126 | 16 | 623 |
| Zhang et al 2014 | 8 | 1.41 | 0.06 | 8 | 1.39 | 0.05 | 55.8 | 16.9 | 1.48 | 10 | Yes | Mix | 101 | 56 | 576 |
| Zhang et al 2014 | 8 | 1.50 | 0.04 | 8 | 1.39 | 0.05 | 55.8 | 16.9 | 1.48 | 15 | Yes | Mix | 101 | 56 | 576 |
| Zhang et al 2014 | 8 | 1.47 | 0.05 | 8 | 1.39 | 0.05 | 55.8 | 16.9 | 1.48 | 20 | Yes | Mix | 101 | 56 | 576 |
| Hall et al 2016 | 4 | 1.32 | 0.34 | 4 | 1.35 | 0.34 | 34.98 | 19.51 | 1.74 | 34.4 | Yes | MP | 101 | 28 | 606 |
| Hall et al 2016 | 4 | 1.34 | 0.34 | 4 | 1.35 | 0.34 | 34.98 | 19.51 | 1.74 | 70.9 | Yes | MP | 101 | 28 | 606 |
| Hall et al 2016 | 4 | 1.60 | 0.34 | 4 | 1.48 | 0.34 | 34.98 | 19.51 | 1.74 | 34.4 | No | MP | 101 | 28 | 606 |
| Hall et al 2016 | 4 | 1.53 | 0.34 | 4 | 1.48 | 0.34 | 34.98 | 19.51 | 1.74 | 70.9 | No | MP | 101 | 28 | 606 |
| Monteiro et al 2017 (i) | 10 | 2.09 | 0.25 | 10 | 1.96 | 0.25 | 59.06 | 15.3 | 1.65 | 100 | No | MP | 0 | 56 | 724 |
| Monteiro et al 2017 (ii) | 8 | 2.03 | 0.28 | 8 | 1.90 | 0.28 | 59.06 | 15.3 | 1.65 | 100 | No | MP | 0 | 56 | 676 |
| Wang et al 2019 | 12 | 1.63 | 0.29 | 12 | 1.52 | 0.29 | 47.21 | 17.49 | 1.63 | 20 | No | MP | 0 | 70 | **676** |
| Shah et al 2020 | 10 | 1.06 | 0.19 | 10 | 1.00 | 0.19 | 49.46 | 14.88 | 1.3 | 15 | Yes | MP | **0** | 60 | 550 |
| Shah et al 2020 | 10 | 1.04 | 0.19 | 10 | 1.00 | 0.19 | 49.46 | 14.88 | 1.3 | 30 | Yes | MP | **101** | 60 | 550 |
| Wang et al 2020 | 12 | 1.44 | 0.13 | 12 | 1.27 | 0.12 | 38.9 | 15.25 | 1.55 | 20 | No | MP | **101** | 56 | **606** |

N, number of cows, M, mean (milk lactose yield kg/day), SD, standard deviation, Cp, crude protein of diet fed to cows (%), NE_L_, net energy of lactation, HS, cows exposed to heat stress or not, parity, parity of the cows, MP, multiparous, PP, primiparous, MIX, Both MP and PP, DIM, days in milk (days), exp duration, duration of the experiment (days), BW, body weight of the cows (kg). The DIM 0 indicates parturition days

Note: Value in the table in blue and bold format were not reported in studies (missing value), we generated these values by random forest algorithm.

Supplementary table 5

Raw data for milk protein yield (kg/day) meta-analysis and variables available for analysis

|  | Betaine | | | Control | | | Variables | | | | | | | | |
| --- | --- | --- | --- | --- | --- | --- | --- | --- | --- | --- | --- | --- | --- | --- | --- |
| Authors and Years | N | M | SD | N | M | SD | Concentrate% | CP % | NE_L_ | Dose mg/day | HS | Parity | DIM | Exp Duration | BW |
| Davidson et al 2008 | 8 | 0.70 | 0.11 | 8 | 0.73 | 0.11 | 59.30 | 17.60 | 1.61 | 45 | No | PP | 28 | 63 | **576** |
| Davidson et al 2008 | 12 | 0.96 | 0.14 | 12 | 0.92 | 0.14 | 59.30 | 17.60 | 1.61 | 45 | No | MP | 28 | 63 | **676** |
| Wang et al 2010 | 20 | 0.84 | 0.06 | 20 | 0.84 | 0.06 | 50.00 | 16.10 | 1.58 | 50 | No | MP | 88 | 15 | 597 |
| Wang et al 2010 | 20 | 0.86 | 0.06 | 20 | 0.84 | 0.06 | 50.00 | 16.10 | 1.58 | 100 | No | MP | 88 | 15 | 597 |
| Wang et al 2010 | 20 | 0.86 | 0.06 | 20 | 0.84 | 0.06 | 50.00 | 16.10 | 1.58 | 150 | No | MP | 88 | 15 | 597 |
| Peterson et al 2012 | 16 | 0.75 | 0.16 | 16 | 0.75 | 0.16 | 63.30 | 18.00 | 1.61 | 25 | No | **MP** | 126 | 16 | 623 |
| Peterson et al 2012 | 16 | 0.75 | 0.16 | 16 | 0.75 | 0.16 | 63.30 | 18.00 | 1.61 | 50 | No | **MP** | 126 | 16 | 623 |
| Peterson et al 2012 | 16 | 0.79 | 0.16 | 16 | 0.75 | 0.16 | 63.30 | 18.00 | 1.61 | 100 | No | **MP** | 126 | 16 | 623 |
| Zhang et al 2014 | 8 | 0.84 | 0.05 | 8 | 0.83 | 0.05 | 55.80 | 16.90 | 1.48 | 10 | Yes | Mix | 101 | 56 | 576 |
| Zhang et al 2014 | 8 | 0.91 | 0.03 | 8 | 0.83 | 0.05 | 55.80 | 16.90 | 1.48 | 15 | Yes | Mix | 101 | 56 | 576 |
| Zhang et al 2014 | 8 | 0.89 | 0.03 | 8 | 0.83 | 0.05 | 55.80 | 16.90 | 1.48 | 20 | Yes | Mix | 101 | 56 | 576 |
| Hall et al 2016 | 4 | 0.76 | 0.19 | 4 | 0.76 | 0.19 | 34.98 | 19.51 | 1.74 | 34 | Yes | MP | 101 | 28 | 606 |
| Hall et al 2016 | 4 | 0.78 | 0.20 | 4 | 0.76 | 0.19 | 34.98 | 19.51 | 1.74 | 70 | Yes | MP | 101 | 28 | 606 |
| Hall et al 2016 | 4 | 0.95 | 0.20 | 4 | 0.80 | 0.19 | 34.98 | 19.51 | 1.74 | 34 | No | MP | 101 | 28 | 606 |
| Hall et al 2016 | 4 | 0.88 | 0.19 | 4 | 0.80 | 0.19 | 34.98 | 19.51 | 1.74 | 70 | No | MP | 101 | 28 | 606 |
| Monteiro et al 2017 (i) | 10 | 1.21 | 0.09 | 10 | 1.14 | 0.09 | 59.06 | 15.30 | 1.65 | 100 | No | MP | 0 | 56 | 724 |
| Monteiro et al 2017 (ii) | 8 | 1.17 | 0.14 | 8 | 1.15 | 0.14 | 59.06 | 15.30 | 1.65 | 100 | No | MP | 0 | 56 | 676 |
| Dunshea et al 2019 | 58 | 0.72 | 1.88 | 58 | 0.68 | 1.88 | **63.00** | **17.60** | **1.61** | 15 | Yes | MP | 0 | 22 | 617 |
| Wang et al 2019 | 12 | 1.03 | 0.20 | 12 | 0.98 | 0.19 | 47.21 | 17.49 | 1.63 | 20 | No | MP | 0 | 70 | **676** |
| Shah et al 2020 | 10 | 0.73 | 0.01 | 10 | 0.69 | 0.01 | 49.46 | 14.88 | 1.30 | 15 | Yes | MP | **0** | 60 | 550 |
| Shah et al 2020 | 10 | 0.71 | 0.01 | 10 | 0.69 | 0.01 | 49.46 | 14.88 | 1.30 | 30 | Yes | MP | **101** | 60 | 550 |
| Wang et al 2020 | 12 | 0.96 | 0.04 | 12 | 0.83 | 0.04 | 38.90 | 15.25 | 1.55 | 20 | No | MP | **101** | 56 | **606** |
| Williams et al 2021 | 12 | 0.73 | 0.07 | 10 | 0.77 | 0.07 | 34.01 | 19.17 | 1.64 | 16 | No | MP | 216 | 35 | 566 |
| Williams et al 2021 | 12 | 0.68 | 0.09 | 10 | 0.71 | 0.09 | 34.01 | 19.17 | 1.64 | 16 | Yes | MP | 216 | 35 | 566 |

N, number of cows, M, mean (milk protein %), SD, standard deviation, Cp, crude protein of diet fed to cows (%), NE_L_, net energy of lactation, HS, cows exposed to heat stress or not, parity, parity of the cows, MP, multiparous, PP, primiparous, Mix, Both MP and PP, DIM, days in milk (days), exp duration, duration of the experiment (days), BW, body weight of the cows (kg). The DIM 0 indicates parturition days.

Note: Value in the table in blue and bold format were not reported in studies (missing value), we generated these values by random forest algorithm.

Supplementary table 6. Raw data for plasma non esterified fatty acids meta-analysis and variables available for analysis

|  | Betaine | | | Control | | | Variables | | | | | | | |
| --- | --- | --- | --- | --- | --- | --- | --- | --- | --- | --- | --- | --- | --- | --- |
| Authors and Years | N | M | SD | N | M | SD | Concentrate% | CP % | NE_L_ | Dose mg/day | HS | DIM | Exp Duration | BW |
| Davidson et al 2008 | 8 | 0.3 | 0.2 | 8 | 0.4 | 0.2 | 59.3 | 17.6 | 1.61 | 45 | No | 28 | 63 | **576** |
| Davidson et al 2008 | 12 | 0.6 | 0.2 | 12 | 0.6 | 0.2 | 59.3 | 17.6 | 1.61 | 45 | No | 28 | 63 | **676** |
| Wang et al 2010 | 20 | 249.0 | 23.7 | 20 | 267.0 | 23.7 | 50 | 16.1 | 1.58 | 50 | No | 88 | 15 | 597 |
| Wang et al 2010 | 20 | 232.0 | 23.7 | 20 | 267.0 | 23.7 | 50 | 16.1 | 1.58 | 100 | No | 88 | 15 | 597 |
| Wang et al 2010 | 20 | 228.0 | 23.7 | 20 | 267.0 | 23.7 | 50 | 16.1 | 1.58 | 150 | No | 88 | 15 | 597 |
| Monteiro et al 2017 (i) | 10 | 300.0 | 117.0 | 10 | 236.0 | 117.0 | 59.06 | 15.3 | 1.65 | 100 | NO | 0 | 56 | 724 |
| Monteiro et al 2017 (ii) | 8 | 353.0 | 113.1 | 8 | 298.0 | 113.1 | 59.06 | 15.3 | 1.65 | 100 | NO | 0 | 56 | 676 |
| Wang et al 2019 | 12 | 243.9 | 71.8 | 12 | 246.6 | 71.8 | 47.21 | 17.49 | 1.63 | 20 | NO | 0 | 70 | **676** |
| Wang et al 2020 | 12 | 185.8 | 117.2 | 12 | 218.0 | 117.2 | 38.9 | 15.25 | 1.55 | 20 | NO | 101 | 56 | **606** |

N, number of cows, M, mean (NEFA), SD, standard deviation, Cp, crude protein of diet fed to cows (%), NE_L_, net energy of lactation, HS, cows exposed to heat stress or not, parity, parity of the cows, MP, multiparous, PP, primiparous, MIX, Both MP and PP, DIM, days in milk (days), exp duration, duration of the experiment (days), BW, body weight of the cows (kg). The DIM 0 indicates parturition days.

Note: Value in the table in blue and bold format were not reported in studies (missing value), we generated these values by random forest algorithm

Supplementary table 7

Raw data for beta hydroxy butyric acid meta-analysis and variables available for analysis

|  | Betaine | | | Control | | | Variables | | | | | | | | |
| --- | --- | --- | --- | --- | --- | --- | --- | --- | --- | --- | --- | --- | --- | --- | --- |
| Authors and Years | N | M | SD | N | M | SD | Concentrate% | CP % | NE_L_ | Dose mg/day | HS | Parity | DIM | Exp Duration | BW |
| Davidson et al 2008 | 8 | 452.0 | 229.1 | 8 | 481.0 | 229.1 | 59.3 | 17.6 | 1.61 | 45 | No | PP | 28 | 63 | **576** |
| Davidson et al 2008 | 12 | 571.0 | 225.2 | 12 | 716.0 | 225.2 | 59.3 | 17.6 | 1.61 | 45 | No | MP | 28 | 63 | **676** |
| Wang et al 2010 | 20 | 729.0 | 36.7 | 20 | 734.0 | 36.7 | 50 | 16.1 | 1.58 | 50 | No | MP | 88 | 15 | 597 |
| Wang et al 2010 | 20 | 720.0 | 36.7 | 20 | 734.0 | 36.7 | 50 | 16.1 | 1.58 | 100 | No | MP | 88 | 15 | 597 |
| Wang et al 2010 | 20 | 717.0 | 36.7 | 20 | 734.0 | 36.7 | 50 | 16.1 | 1.58 | 150 | No | MP | 88 | 15 | 597 |
| Monteiro et al 2017 (i) | 10 | 98.9 | 15.2 | 10 | 87.4 | 15.2 | 59.06 | 15.3 | 1.65 | 100 | NO | MP | 0 | 56 | 724 |
| Monteiro et al 2017 (ii) | 8 | 92.2 | 17.7 | 8 | 81.6 | 17.7 | 59.06 | 15.3 | 1.65 | 100 | NO | MP | 0 | 56 | 676 |
| Wang et al 2019 | 12 | 812.7 | 320.6 | 12 | 802.7 | 320.6 | 47.21 | 17.49 | 1.63 | 20 | NO | MP | 0 | 70 | 676 |

N, number of cows, M, mean (BHBA), SD, standard deviation, Cp, crude protein of diet fed to cows (%), NE_L_, net energy of lactation, HS, cows exposed to heat stress or not, parity, parity of the cows, MP, multiparous, PP, primiparous, MIX, Both MP and PP, DIM, days in milk (days), exp duration, duration of the experiment (days), BW, body weight of the cows (kg). The DIM 0 indicates parturition days.

Note: Value in the table in blue and bold format were not reported in studies (missing value), we generated these values by random forest algorithm

**
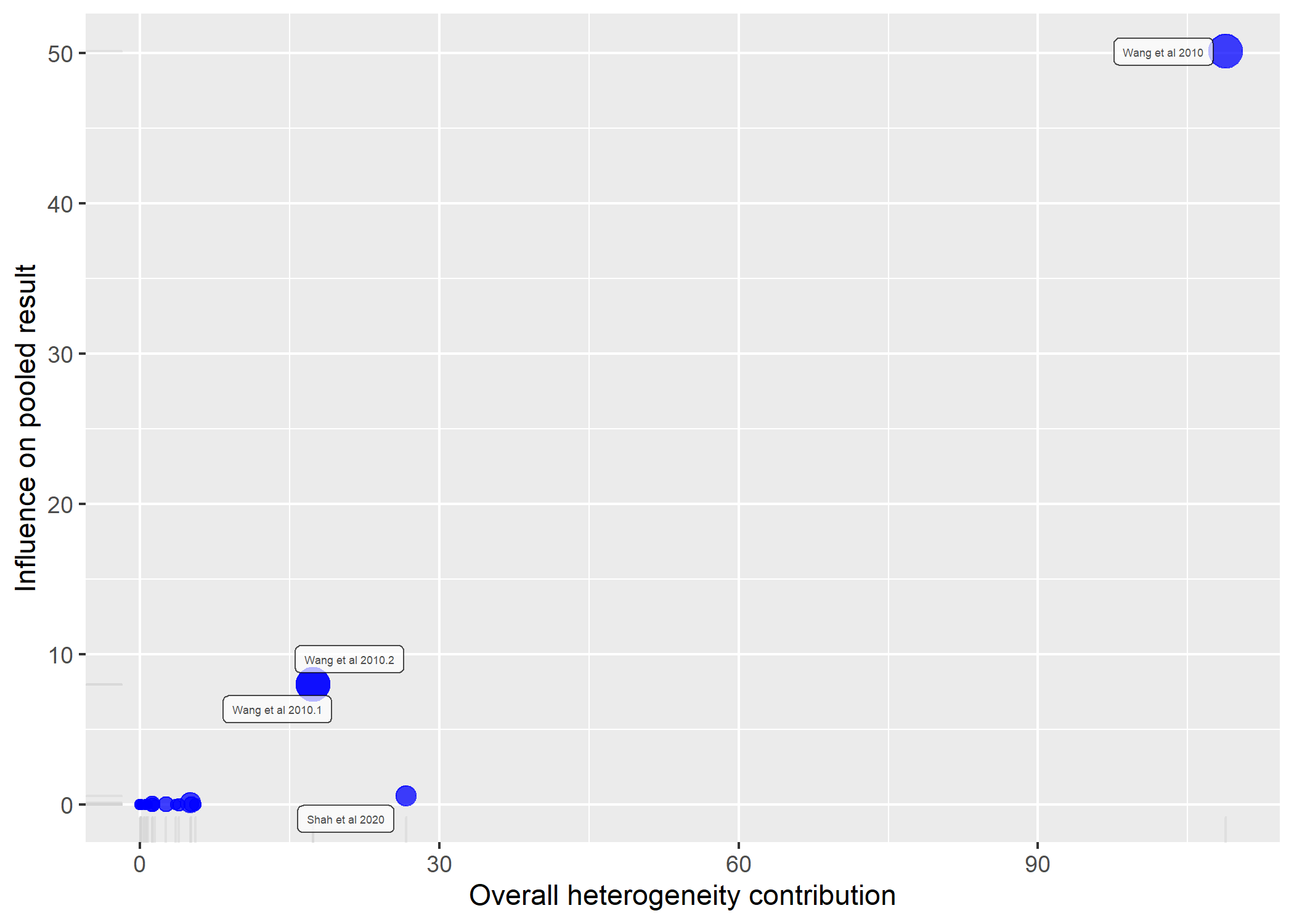
**

**Supplementary figure 1.** The Baujat diagnostics plot to visualize the heterogeneity contribution of each study included in milk production meta-analysis


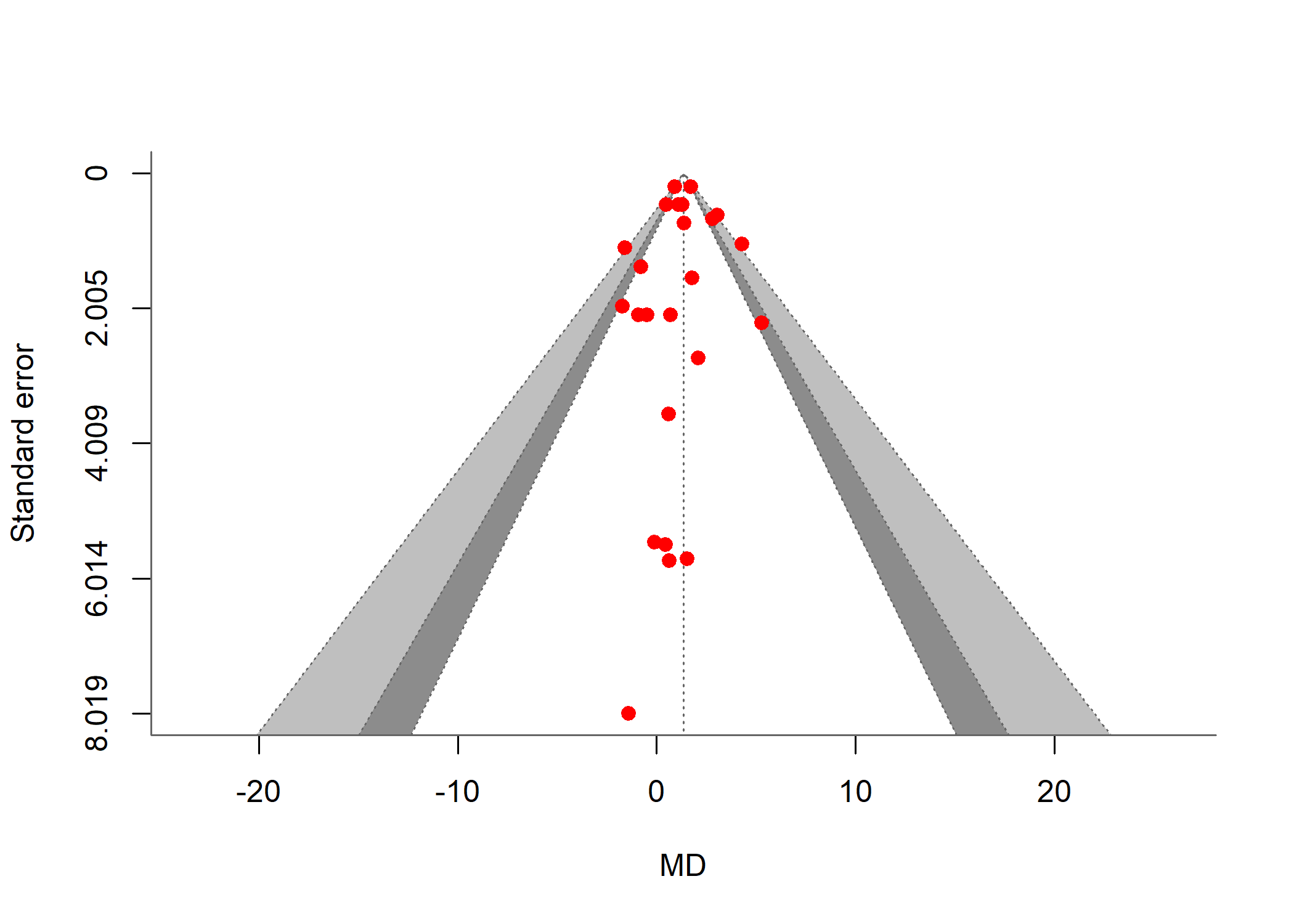


**Supplementary figure 2.** The contour enhanced funnel plot shows the publication bias, the study's symmetrical distribution of effect sizes around standard error shows no evidence of bias in the meta-analysis of energy corrected milk production (kg). DIM, days in milk, CP, dietary protein, Con= concentrate portion of the diet, exp duration= duration of the experiment, BW=cows' body weights, dose= betaine supplementation, and parity= cows' parity


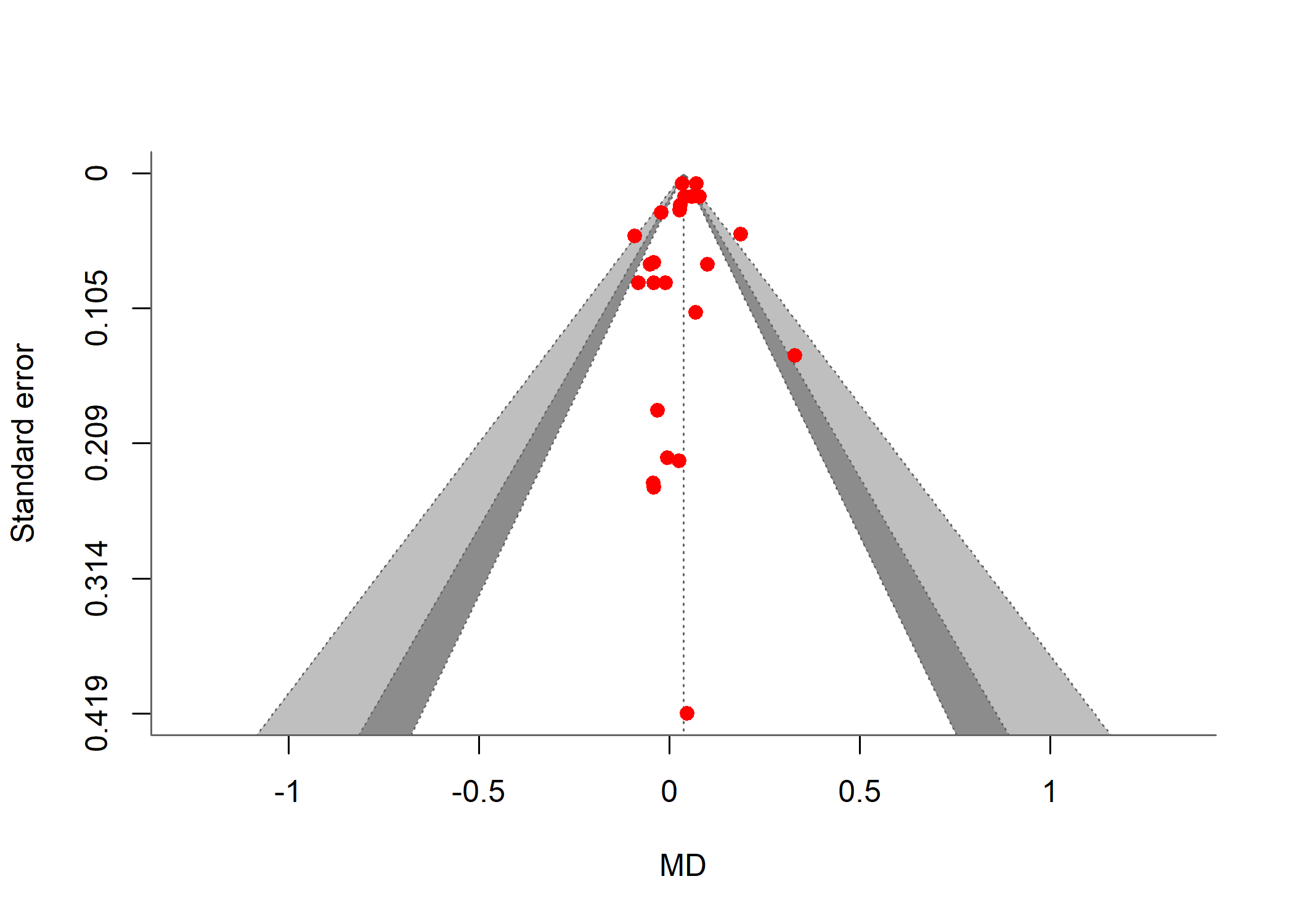


**Supplementary figure 3.** The contour enhanced funnel plot shows the publication bias, the study's symmetrical distribution of effect sizes around standard error shows no evidence of bias in the meta-analysis of milk fat yield (kg/day). DIM, days in milk, CP, dietary protein, Con= concentrate portion of the diet, exp duration= duration of the experiment, BW=cows' body weights, dose= betaine supplementation, and parity= cows' parity


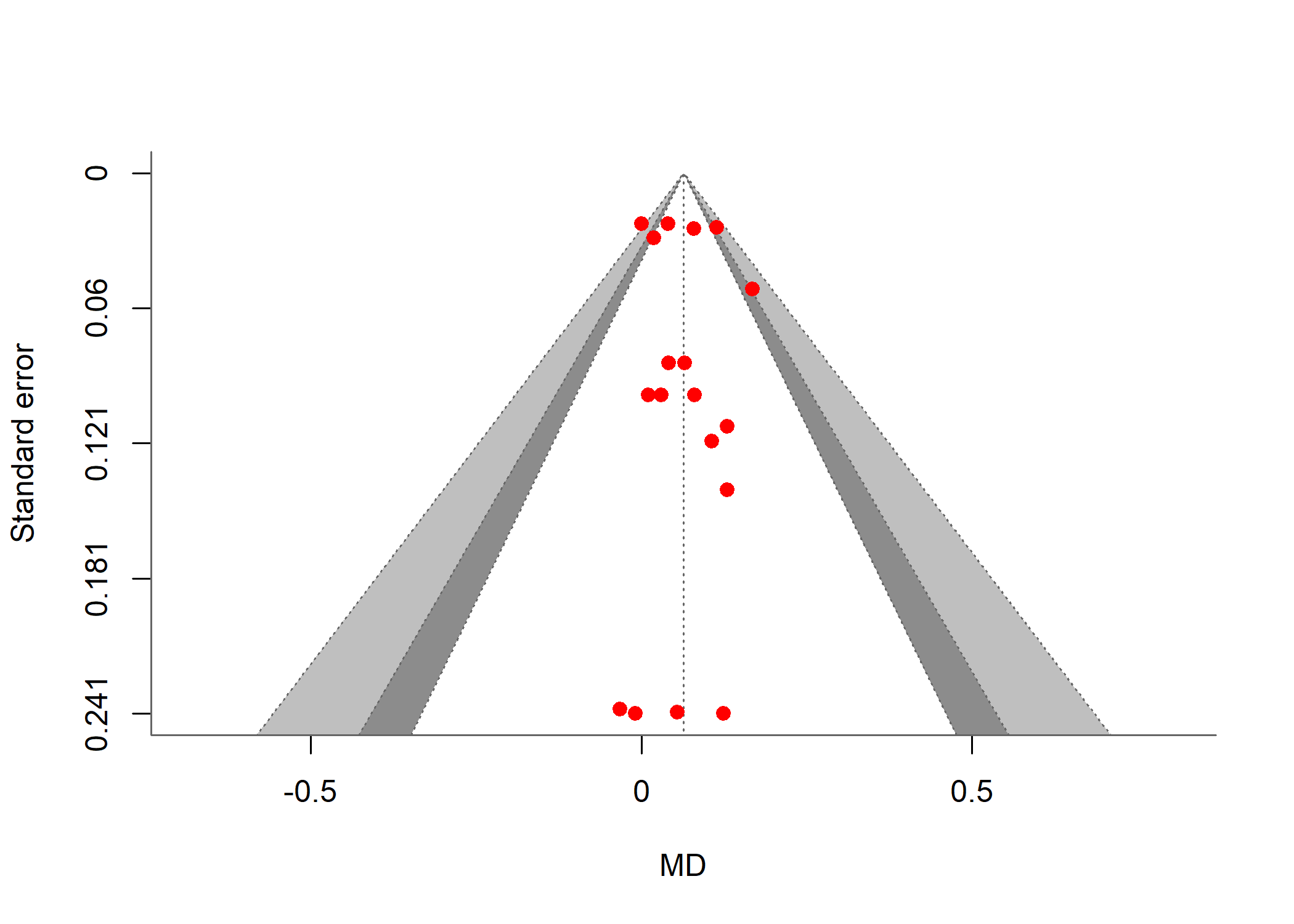


**Supplementary figure 4.** The contour enhanced funnel plot shows the publication bias, the study's symmetrical distribution of effect sizes around standard error shows no evidence of bias in the meta-analysis of milk lactose yield (kg/day). DIM, days in milk, CP, dietary protein, Con= concentrate portion of the diet, exp duration= duration of the experiment, BW=cows' body weights, dose= betaine supplementation


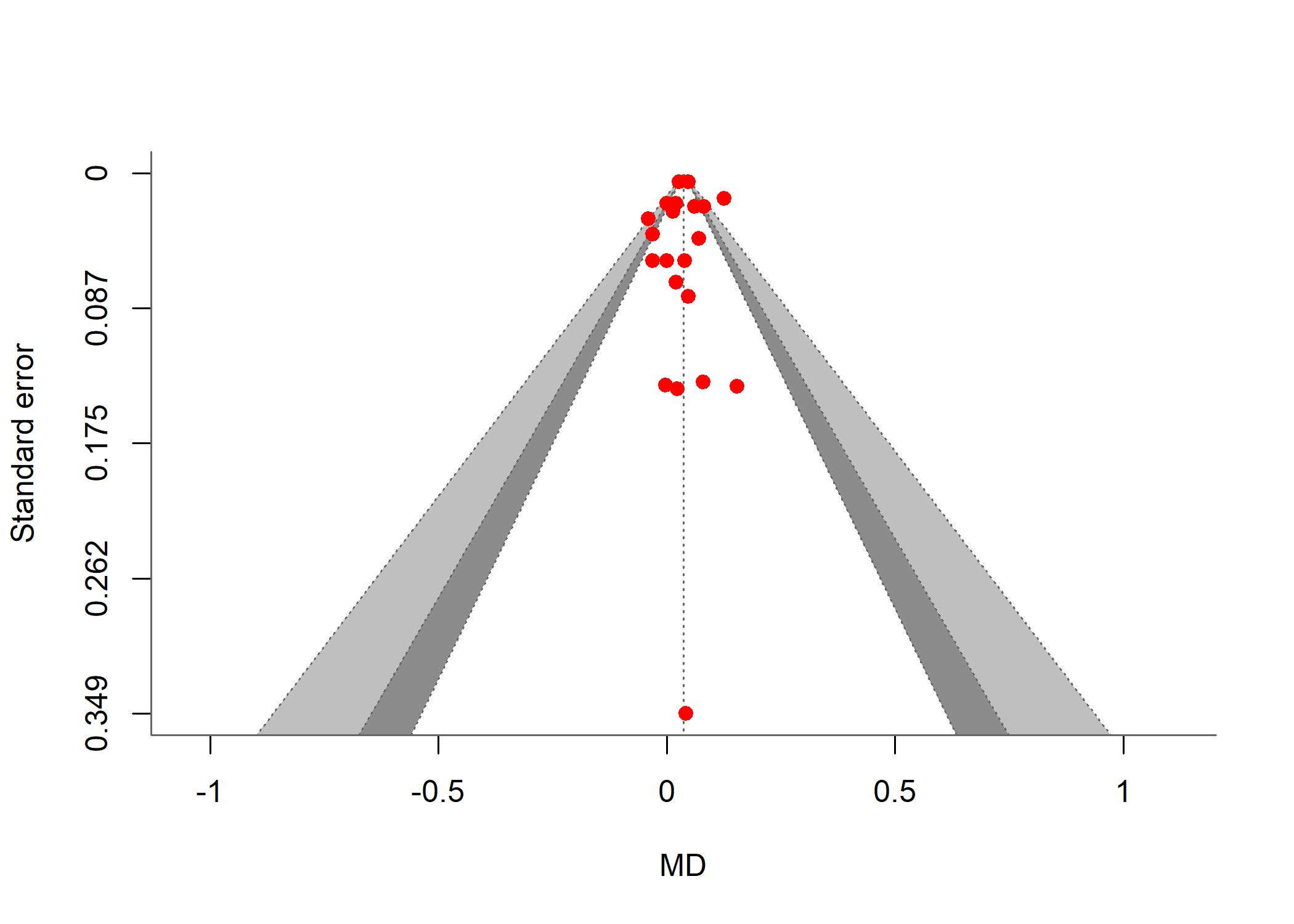


**Supplementary figure 5**. The contour enhanced funnel plot indicates the publication bias, the symmetrical distribution of effects size around standard error of the study represents no evidence of bias in milk protein yield (kg/day) meta-analysis
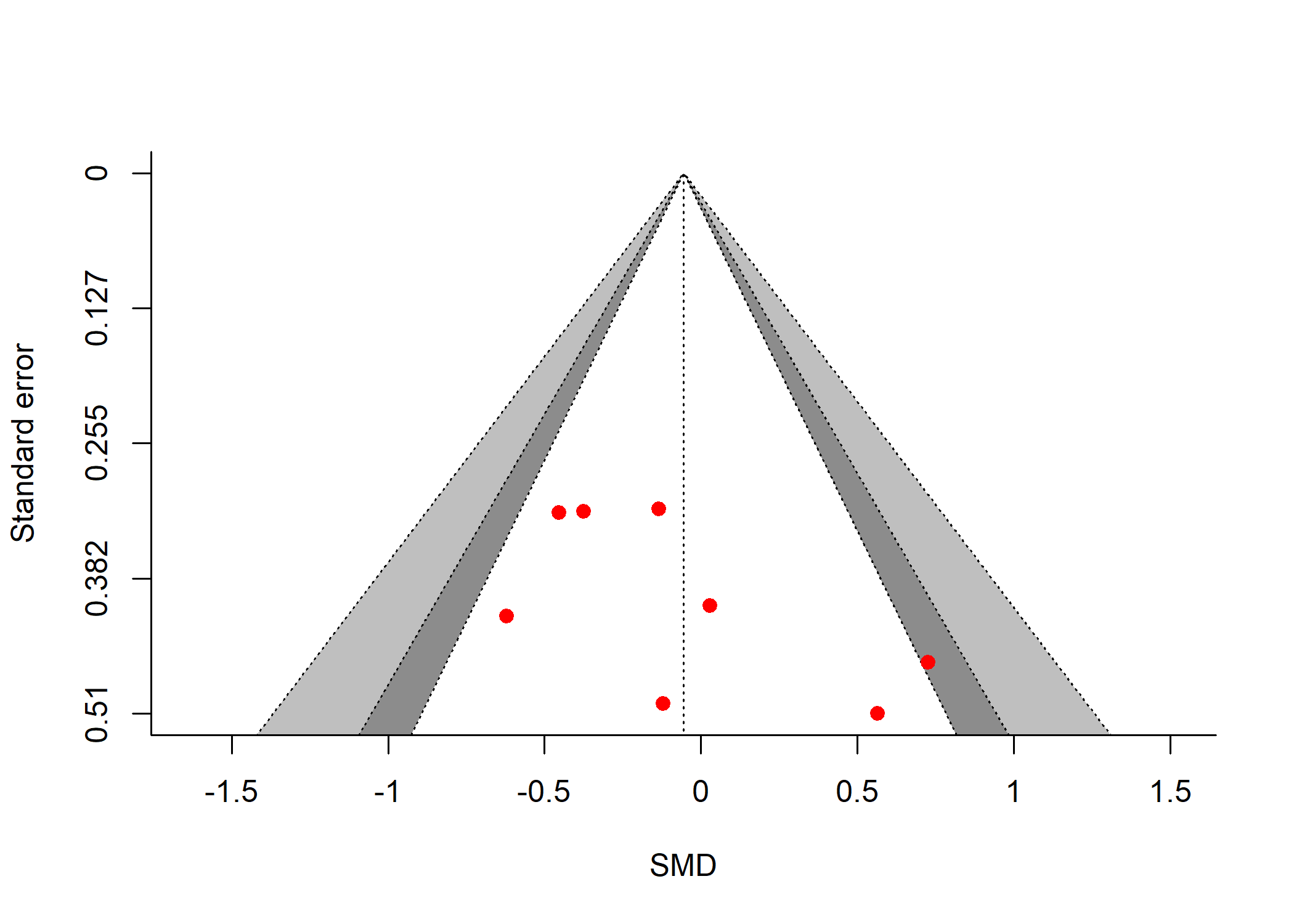


**Supplementary figure 6**. The contour enhanced funnel plot indicates the publication bias, the symmetrical distribution of effects size around standard error of the study represents no evidence of bias in BHBA meta-analysis.
